# Gonadotropins differentially regulate testicular cell adhesion and junctional complexes during flatfish spermiogenesis through the oxytocin and relaxin signaling pathways

**DOI:** 10.3389/fcell.2025.1574690

**Published:** 2025-06-02

**Authors:** Noelia López-Fortún, Jose Vicente Roig-Genovés, Ignacio Giménez, Joan Cerdà, François Chauvigné

**Affiliations:** ^1^ Institute of Marine Sciences, Spanish National Research Council (CSIC), Barcelona, Spain; ^2^ Institute of Biotechnology and Biomedicine (IBB), Universitat Autònoma de Barcelona, Barcelona, Spain; ^3^ Rara Avis Biotec, S.L., Valencia, Spain

**Keywords:** teleost, spermatogenesis, blood-testis barrier, cell junctions, adherens junctions, endocrine control

## Abstract

**Introduction:**

The molecular mechanisms regulating teleost semicystic spermatogenesis remain largely unknown. In the flatfish Senegalese sole (*Solea senegalensis*), haploid round spermatids released into the lumen of the seminiferous tubules resume spermiogenesis (the differentiation of germ cells into spermatozoa) in response to the luteinizing hormone (Lh). However, how the spermatids detach from Sertoli cells and how Lh crosses the blood-testis barrier (BTB) are yet to be determined.

**Methods:**

Here, we used an RNA-seq transcriptomic analysis of the testis from sole males treated with recombinant follicle stimulating hormone and Lh (rFsh and rLh, respectively).

**Results:**

This analysis reveals that both gonadotropins differentially downregulate a number of transcripts potentially encoding cell-cell junction and adhesion proteins, as well as components of the Oxytocin (Oxt) and Relaxin (Rln) signaling pathways. *In situ* hybrizidation and immunolocalization experiments confirmed the formation of adherens, gap, and tight junctions between Sertoli cells, and between Sertoli cells and spermatids. Using these methods, we also verified the expression of Oxt and Rln peptides and their cognate receptors in these cells. Further *in vitro* assays using testicular explants incubated with Oxt, Rln and inhibitors of their receptors, combined with rFsh or rLh, showed that the gonadotropic-induced transcriptional repression of cell junction and adhesion genes in the seminiferous epithelium, particularly by Lh, was largely mediated by the downregulation of Oxt and Rln signaling.

**Discussion:**

These data suggest that the Oxt- and Rln-mediated gonadotropic disruption of the BTB and Sertoli cells-spermatid junctions in the sole testis facilitates spermatid release and Lh paracellular transport into the seminiferous lumen during spermiogenesis.

## Introduction

Most teleost species exhibit cystic and cyclical spermatogenesis, where spermatozoa differentiate from spermatids (spermiogenesis) embedded in the somatic Sertoli cells forming the seminiferous tubules. Spermatozoa subsequently detach from Sertoli cells and are released into the lumen of the tubules (spermiation) (Schulz et al., 2023). Unlike most teleosts, the flatfish Senegalese sole (*Solea senegalensis*) displays a semi-cystic and asynchronous pattern of germ cell development Its testis is divided into two main regions: the outer cortical region and the inner medullary region. The seminiferous tubules originate in the cortex and contain germ cells supported by Sertoli cells. This type of testis is classified as having restricted spermatogenic tubules. Early-stage cysts containing spermatogonia are localized peripherally, with spermatogenesis progressing inward along the tubule. In the medulla, cysts begin to open and spermatids, initially enclosed in Sertoli cells, are released. As a result, spermiogenesis occurs within the lumen of the tubules, a process known as semi-cystic spermatogenesis. The resulting sperm are transported toward the efferent ducts, where they are collected and stored. (Uribe et al., 2014; García-López et al., 2005; Schulz et al., 2023). This unusual mechanism of spermiogenesis and spermiation in sole provides an excellent experimental model to investigate the signaling pathways underlying the transport of germ cells across the seminiferous epithelium in teleosts. In particular, the role of cell adhesion proteins during this process remains largely unknown.

In mammals, the detachment of spermatids from Sertoli cells during spermiation relies on the modulation of cell adhesion molecules (CAMs), which form adherens junctions (e.g., N-cadherin), gap junctions (e.g., connexin-43) and tight junctions (e.g., occludins and claudins). These adhesion and junctional structures also contribute to the formation of the blood-testis barrier (BTB) at the Sertoli cell-cell interface, which protects the developing germ cells during spermatogenesis (Kopera et al., 2010; McCabe et al., 2010; Piprek et al., 2020; Luaces et al., 2023). Ectoplasmic specializations (ES), which are testis-specific actin-based atypical anchoring junctions, are associated to the Sertoli cell tight junctions. Tey occur at the interface between Sertoli cells at the BTB (basal ES) and at the interface between Sertoli cells and developing spermatids (apical ES) (Wong et al., 2008a). These complexes are formed by the interaction of different proteins, such as nectin-3, ezrin, α3β3γ3-laminin and α6β1-integrin (Gungor-Ordueri et al., 2014; O’Donnell et al., 2011; Wong et al., 2008b). During spermiation, the BTB temporarily opens due to the downregulation of CAMs and junctional proteins, allowing mature spermatids to cross the germinal epithelium. Simultaneously, disorganization of the apical ES allows the release of spermatids into the lumen of the seminiferous tubules. However, the endocrine regulation of the testicular cell adhesion and junctional complexes during spermiation is not yet well understood (Lui and Cheng, 2012). The pituitary gonadotropins, follicle-stimulating and luteinizing hormones (FSH and LH, respectively) may regulate these processes indirectly via steroid hormones produced by Leydig cells (O’Shaughnessy, 2014; Bhattacharya et al., 2023). When testosterone acts through the androgen receptor, spermiation is blocked due to persistent focal adherens junctions in the BTB (Smith and Walker, 2014; Kumar et al., 2017). Non-classical testosterone signaling via activation of the tyrosine kinase Src and mitogen-activated protein kinase/ERK pathway promotes germ cell adhesion to Sertoli cells. In contrast, FSH counteracts this androgenic action and contributes to spermatid release (Shupe et al., 2011). Recent studies have also highlighted the roles of oxytocin (OXT) and relaxin (RLN) peptide hormones in testicular cell adhesion, where OXT aids seminiferous tubule contraction, while RLN promotes cell contact by regulating β-catenin expression (Kumar et al., 2018; Assinder et al., 2002; Pimenta et al., 2015).

In teleosts, the BTB is formed by Sertoli cells surrounding spermatids and involves tight and gap junctions, while apical ES are apparently absent (Batlouni et al., 2009; Leal et al., 2009). However, as in mammals, spermatid release is also associated with the opening of the Sertoli cells, potentially aiding germ cell nourishment through seminal fluid secretion (Loir et al., 1995). The mechanisms that controlling BTB permeability in fish are less understood than in mammals. However, hormones such as estradiol and insulin-like 3 (Insl3), a member of the insulin/relaxin hormone superfamily, have been shown to modulate connexin-43 expression during spermatogenesis and spermiation (Zheng et al., 2019; Crespo et al., 2021). Moreover, the presence of Oxt and Rln peptides and receptors in fish testicular tissues suggests the involvement of these signaling pathways in the regulation of cell adhesion as in mammals (Crespo et al., 2021; Lema, 2010; Wilson et al., 2009; Mennigen et al., 2022; Yang et al., 2020). In the Senegalese sole, the specific regulation of CAMs and junctional proteins during spermiogenesis may play a key role, since during this process, mature spermatids expressing Lh receptor (Lhcgrba)are released into the lumen of the seminiferous tubules where they differentiate to spermatozoa in response to the Lh (Chauvigné et al., 2014a). However, the mechanisms by which spermatids detach from Sertoli cells, as well as those allowing the circulating Lh to traverse the BTB to reach the spermatids free in the lumen, are completely unknown.

The aim of the present study was to address the potential regulatory roles of Fsh and Lh in modulating the BTB during spermiogenesis and spermiation in Senegalese sole. For this purpose, we conducted an RNA-seq transcriptomic analysis of the testis of males treated with recombinant gonadotropins (rFsh or rLh) for 6 weeks. Combined with the cellular localization studies and *in vitro* experiments on testicular explants, our results show that both gonadotropins modulate the BTB and Sertoli cell-spermatid junctions dynamics through differential regulation of the Oxt and Rln signaling pathways.

## Materials and methods

### Fish and experimental design

Two-year-old male Senegalese sole, F1 generation offspring from wild-caught parents, were acquired from the commercial supplier Stolt Sea Farm S.A. (Spain) and maintained at the Institute of Agrifood Research and Technology (IRTA) research facilities in Sant Carles de la Ràpita (Spain), as described previously (Chauvigné et al., 2017). The experiment was conducted over 6 weeks from November to January to examine the effect of rFsh and rLh on testicular development. Males (347 ± 9 gr) received intramuscular injections once a week with saline (n = 8), rFsh (18 μg/kg, n = 8) or rLh (18 μg/kg, n = 8). At time zero and after 6 weeks of treatment, fish were anesthetized with 60 mg/L tricaine methanesulfonate (MS-222; Sigma-Aldrich), weighed. A 0.5–1 mL blood sample was collected from the caudal vein using a syringe pre-coated with 0.5 M EDTA pH 8. The blood was transferred into a tube containing 5 μL EDTA, centrifuged at 3000 *g* for 15 min at 4°C, and the plasma aliquoted and stored at −80°C. In some trials, fish were immediately euthanized by decapitation, and the testes were excised to calculate the gonadosomatic index (GSI; testes weight/fish weight × 100). The right testis was divided into two parts, and separately prepared for histology and immunohistochemistry. The left testis was cut into small pieces (∼20 mg each), deep-frozen in liquid nitrogen, and stored at −80°C. The animal care and sample collection procedures were conducted in compliance with protocols approved by the Ethics Committee (EC) from IRTA following the European Union Council Guidelines (86/609/EU).

### Antibodies and reagents

Single-chain Senegalese sole rFsh and rLh were produced by Rara Avis Biotec (Valencia, Spain) as described previously (Chauvigné et al., 2017). Mouse monoclonal antibody against 5-bromo-2′-deoxyuridine (BrdU) was obtained from the Developmental Studies Hybridoma Bank (G3G4; University of Iowa). The connexin 46/GJA3 antibody was from Bio-Techne (NBP1-59197), the SCRIB (PA5-79959) and TJP2 (BS-4844R) polyclonal antibodies were from Thermo Fisher Scientific, and the anti-PARD3 antibody (HPA030443-25UL), anti-CTNNB1 antibody (HPA029159-25UL), and anti-RAB5C antibody (HPA003426-25UL) were from Sigma-Aldrich. The Oxt antibody was generously provided by Prof. Olivier Kah (INSERM-Université de Rennes 1, France. Specific antibodies against Senegalese sole Fsh receptor a (Fshra) and Lh receptor ba (Lhcgrba) have been characterized previously (Chauvigné et al., 2012). The secondary antibodies used included Alexa Fluor 488-conjugated goat anti-rabbit and anti-mouse IgG (A-11008 and A-11029, respectively; Life Technologies Corp.), and horseradish peroxidase-conjugated goat anti-rabbit IgG (sc-2004; Santa Cruz Biotechnology, Inc.). All other reagents and kits were from Life Technologies Corp. unless specified otherwise.

### Determination of hormone plasma levels

Plasma levels of the androgen 11-ketotestosterone (11-KT) were measured using a commercial enzyme immunosorbent assay (EIA; Cayman Chemical Company) as described previously (Chauvigné et al., 2012). Free steroids were extracted from plasma (50 μL) in methanol, and the resulting pellet was diluted 1:500 in EIA buffer (0.1 M K_2_HPO_4_/KH_2_PO_4_, 1.54 mM sodium azide, 0.4 M NaCl, 1 mM EDTA, and 0.1% BSA, pH 7.4). All samples were analysed in duplicate, and a separate standard curve was generated for each EIA plate. The plasma levels of Fsh and Lh were determined using a homologous custom-made EIA as previously described (Chauvigné et al., 2015; 2016).

### Histological analysis

Testis biopsies were fixed, sliced and sections stained with hematoxylin and eosin as described previously (Chauvigné et al., 2017). Somatic (Sertoli and Leydig cells) and germ cells were identified following the criteria of García-López et al. (2005). The number of each type of germ cell in the cortical and medullar regions of the testis was counted in 10 seminiferous tubules of 5–10 different areas of the cortex and medulla for each fish using the NIS-element AR 4.30.02 software (Nikon). With these data, the percentage of germ cells in each tubule was calculated.

### RNA extraction, library construction, and sequencing

Total RNA from the testis was extracted using the RNeasy Plus Mini Kit (Qiagen). The RNA purity and concentration were assessed using the NanoDropVC 2000, and RNA size distribution profiles were analysed using the Agilent 2100 Bioanalyzer. RIN values were comprised between 8.7 and 9.8. For library construction (n = 4 for each group: saline, rFsh and rLh), the SMARTseq2 protocol was employed, with some modifications. The libraries were sequenced on a NovaSeq 6000 S1 sequencing system (Illumina) in paired-end mode with 100 bp paired-end reads length. The resulting reads were processed for image analysis, base calling, and quality scoring using the manufacturer’s software, Real Time Analysis (RTA 1.18.66.3), followed by the generation of FASTQ sequence files by CASAVA 1.8.

### Differential expression analysis

The RNA-seq raw reads were pre-processed for trimming adapters and quality filtering (reads below a Q30 Phred score were discarded) using Cutadapt software v3.4 (Martin, 2011). Read quality was assessed before and after adapter/quality trimming using FastQC software v0.11.9 (Wingett and Andrews, 2018). Subsequently, reads were aligned against the *S. senegalensis* reference genome (GCF_019176455.1) using Subread software v2.0.1, with all samples showing >70% of successfully assigned reads. Annotation of aligned reads was performed via R software (v4.0.4) and the RSubread package v2.2.6 (Liao et al., 2013) at the gene level using the GCA_019176455.1 gff file with default parameters. Genes with fewer than 50 reads in all samples were excluded from differential expression analysis. Principal component analysis (PCA) was performed after trimmed mean of M values (TMM) count normalization to explore the global gene expression pattern in the samples. Differential expression analysis was performed using DESeq2 v1.30.1 (Love et al., 2014) with default parameters. Pairwise differential expression analyses were performed between treatment groups (rFsh vs. Saline, rFsh vs. rLh, rLh vs. Saline), with genes having an adjusted *p*-value <0.05, considered to show statistically significant differential expression.

### Functional enrichment analysis and *de novo* transcriptome assembly

Human (*Homo sapiens*) homologs of the sole differentially expressed genes (DEGs) were used for functional enrichment analysis, given the more extensive and robust functional information available for human genes compared to *S. senegalensis*. The identifiers of the DEGs in *S. senegalensis* (Gene ID) were converted to RefSeq Protein Accession using the bioDBnet platform. Using these identifiers, a protein-protein search (Protein-Protein BLAST software v2.12.0) (Camacho et al., 2009) was performed against the human proteome (GRCh38.p14) with the following parameters: e-value 1e-10 as a threshold. ENSEMBL protein IDs of human homologous genes were obtained, selecting the highest alignment score, considering matches with the lowest e-value and higher protein coverage. Visualization of DEGs was done through heatmaps, Venn diagrams, and volcano plots. For the characterization and identification of transcripts of high interest, a *de novo* transcriptome assembly was performed using Trinity v2.12.0 (Haas et al., 2013), with default parameters for a pool of 5 samples.

### Gene ontology (GO), and pathway and interactome analyses

For a deeper understanding of the identified DEGs, GO and KEGG enrichment and signaling pathway analyses of DEGs were performed using the clusterProfiler R package v4.2.2. (Yu et al., 2012). Volcano plots were created using the VolcaNoseR software (http://goedhart.shinyapps.io/VolcaNoseR/) and heatmaps with HeatMapper (http://www.heatmapper.ca/expression/). The GO enrichment and pathway analyses were performed using the PANTHER classification system. GO terms and pathways with an FDR <0.05% were considered significant, and KEGG terms with an adjusted p-value <0.05 were considered to show statistically significant enrichment. Additionally, functional category classifications were carried out manually using the Uniprot database and QuickGO browser. Interactome analyses were conducted using the STRING database, and plots were generated using Cytoscape. In some instances, selected transcripts were mapped using the KEGG pathway database and WikiPathways using Kobas software (http://kobas.cbi.pku.edu.cn/). The plots were generated with the “ggplot2” R package (https://ggplot2.Tidyverse.org).

### Real-time quantitative PCR (RT-qPCR)

The expression levels of specific genes were measured by qRT-PCR using testis samples from the hormone-treated fish but different from those employed for RNA-seq. Total RNA was isolated from the testes using the GenElute™ Mammalian Total RNA Miniprep Kit (Sigma-Aldrich), treated with DNase I, and 1 μg of total RNA was reverse transcribed using 0.5 μg oligo (dT)17, 1 mM dNTPs, 40 IU RNAse inhibitor, and 10 IU SuperScript II (Life Technologies Corp.) for 1.5 h at 42°C. The RT-qPCR was performed in a final volume of 10 μL with 5 μL of iTaq Universal SYBR Green Supermix (BioRad, #1725120), 1 μL of diluted cDNA (1:5 in sterile mQ water), and 0.5 μM of each specific forward and reverse oligonucleotide primers (Supplementary Table S1). The reference gene used was alpha tubulin. Each sample was analyzed in duplicate on 384-well plates using the C1000 Touch Thermal Cycler with the optical modules CFX384 (Biorad, LLEB, UAB). The amplification protocol included an initial denaturation and activation phase at 50°C for 2 min and 95°C for 10 min, followed by 40 cycles of 95°C for 15 s and 63°C for 1 min. After the amplification step, a melting curve analysis was conducted at 95°C for 15 s, 60°C for 15 s, and 95°C for 15 s. Variations in gene expression in testicular explants were calculated as fold-changes relative to the saline group using the 2^−ΔΔCt^ method (Livak and Schmittgen, 2001).

We validated the RNAseq data by comparing group-wise z-score changes with corresponding qPCR results (Supplementary Figure S1). The resulting graph a strong correlation between the two methods, with an *R* ^2^ value ranging from 0.7 to 0.8.

### Immunofluorescence microscopy

Testis samples were incubated with 10 μM BrdU for 1 h in L-15 without phenol red supplemented with 10 mM HEPES, 0.5% BSA (Sigma-Aldrich), 0.4 mg/mL Fungizone (Sigma-Aldrich), and 200 μg/mL penicillin/streptomycin, washed twice in L-15, and fixed in 4% paraformaldehyde (PFA; Sigma-Aldrich) for 6 h at room temperature. After washing, dehydration, and embedding in paraplast, sections of 7 μm thickness were attached to UltraStick/UltraFrost Adhesion slides and rehydrated before permeabilization. For the BrdU antibody, sections were permeabilized with boiling citrate at 0.01M and pH 6 for 5 min, repeated 3 times. After cooling to room temperature, the slides were washed in phosphate buffer solution (PBS; 20 mM Na_3_PO_4_, 500 mM NaCl, pH 7.4) and subjected to a second permeabilization step in 2 N HCl for 20 min at 37°C, then quickly washed in PBS and exposed to 0.07 M NaOH for 10 min. Sections were rinsed again in PBS and incubated with 0.2% Triton X-100 for 15 min at room temperature. For the other antibodies, the permeabilization was done in boiling 0.01 M citrate for 30 min and after permeabilization, sections were blocked in 5% goat serum and 0.1% BSA in PBS with 0.02% triton X-100 for 1 h before incubation with the antibody (1:300 in PBS) overnight at 4°C. Alexa Fluor 488-coupled goat anti-mouse IgG (for BrdU antibody), or Cy3-coupled sheep anti rabbit IgG (for all other antibodies), were added for 1 h at room temperature. After washing, the cell nuclei were counterstained with DAPI (D9564, 1:5000 in PBS for 5 min), while membranes were revealed with Alexa Fluor 647-conjugated wheat germ agglutinin (WGA, W32466, 1:3000 in PBS for 5 min). Slides were finally mounted using fluoromount aqueous anti-fading medium (Sigma-Aldrich), and examined and photographed with a Zeiss Axio Imager Z1/ApoTome fluorescence microscope (Carl Zeiss, Jena, Germany).

### *In situ* hybridization (ISH)

The protocol employed for ISH followed that described by Chauvigné et al. (2014b) with some modifications. Hybridization was conducted overnight at 50°C using 2 μg/mL DIG-labelled riboprobes corresponding to specific sequences of the cDNAs amplified by PCR (see primers in Supplementary Table S2). After hybridization, sections were washed four times in 2× SSC, followed by RNase A treatment (10 μg/mL in 2 × SSC) for 30 min at 37°C. The sections were washed again in 2 × SSC for 15 min at 50°C, twice in 0.2 × SSC Sections were mounted with Fluoromount aqueous mounting medium and images were captured using a Leica light microscope DMR.

### *In vitro* culture of testis explants

The testes from three males were cut into pieces assuring the presence of both cortical and medullar regions. The testicular explants were incubated in triplicate for each treatment in L-15 medium without phenol red supplemented with gentamycin (100 μg/mL), 0.2% bovine serum albumin (BSA) and 10 mM Hepes, at pH 7.5. The treatments included incubation with 100 ng/mL of rFsh or rLh, 100 ng/mL RLN2 (Sigma-Aldrich; #SRP3147) or 100 ng/mL OXT (PeptideSynthetics, Peptide Protein Research Ltd.; Ferré et al., 2023), or a combination of rFsh or rLh with RLN2 or OXT. The explants incubated with RLN2 or OXT were also treated with 10 µM of the RLN receptor antagonist AT-001 (MedKoo, CAS#2099681-43-1) or 10 µM of the OXT receptor antagonist L-371,257 (Tocris, #2410) for 1 h prior to stimulation with the corresponding hormone. The control explants were incubated with the same concentration of drug vehicle (0.1% DMSO). Testicular explants were incubated for 24 h at 16°C, followed by deep frozen of the explants in liquid nitrogen and storage at −80°C prior to RNA extraction.

### Statistical analyses

Data are expressed as mean ± standard error of the mean (SEM). Data on RT-qPCR and hormonal levels, cell types and GSI were statistically analyzed by one-way ANOVA, followed by the Tukey’s multiple comparison test, or by the non-parametric Kruskal–Wallis test and further Dunn’s test for nonparametric *post hoc* comparisons. The *in vitro* experimental data were statistically analyzed by the two-tailed unpaired Student’s t-test, or by the nonparametric Mann Whitney test when variances were not equal. Statistical analyses were carried out using GraphPad Prism v9.4.1 (681) software (GraphPad Software). In all cases, differences were considered significant at *P* < 0.05.

## Results

### Effects of recombinant gonadotropins on spermatogenesis

**FIGURE 1:**
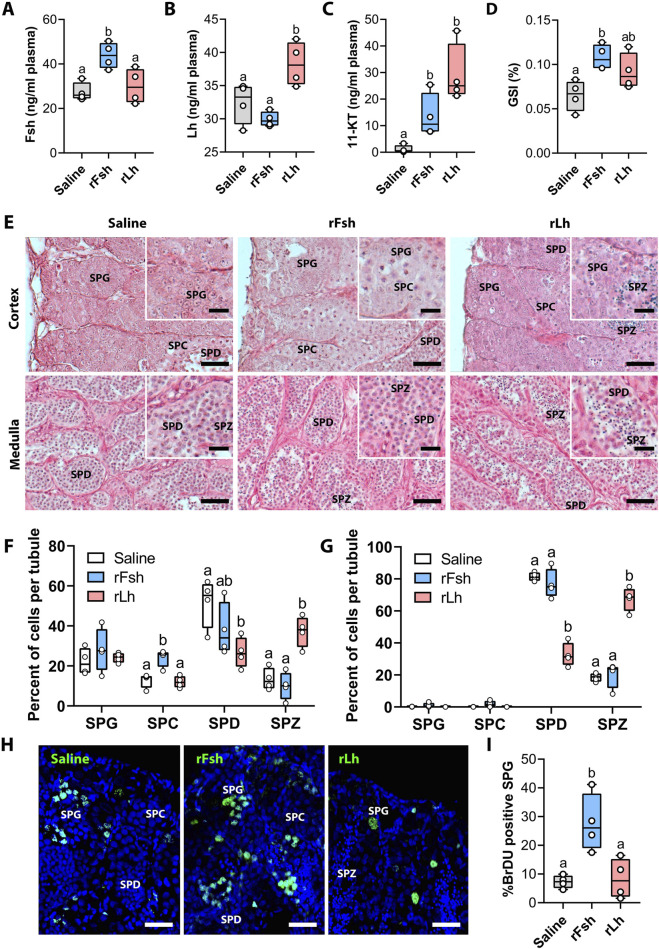
Circulating hormone levels, gonad weight and proliferation of testicular germ cells in males treated with rFsh or rLh. **(A–C)** Plasma levels of Fsh **(A)**, Lh **(B)** and 11-KT **(C)** under the different treatments (saline, rFsh or rLh) for six consecutive weeks, measured 1 week after the last injection. **(D)** Gonadosomatic index (GSI), calculated as gonad mass relative to total body mass, under the different hormone treatments. **(E)** Representative photomicrographs of histological sections from the testicular cortical and medullar regions. Scale bars, 10 μm (Inset, 3 µm). SPG, spermatogonia; SPC, spermatocyte; SPD, spermatid; SPZ, spermatozoa. **(F,G)** Percentage of germ cells in the cortical **(F)** and medullar **(G)** regions of the testis from males treated with rFsh or rLh. **(H)** Immunostaining of BrdU, highlighting proliferating SPG (in green), which indicates active cell division under the different treatments. Scale bars, 30 µm **(I)** Quantification of BrdU-positive SPG under the different treatments. In A-D, F, G and I, data are presented as box and whisker plots/scatter dots with horizontal line (inside box) indicating median and outliers (n = 4 fish, white dots), and were statistically analyzed by one-way ANOVA followed by the Tukey’s multiple comparison test. Bars with different superscript are significantly different (*P* < 0.05).

Male sole were injected intramuscularly with saline (controls) or 18 μg/kg of rFsh or rLh for six consecutive weeks. To validate the efficiency of the rFsh and rLh treatments to enhance spermatogenesis and spermiation, we performed ELISA assays for Fsh, Lh, and 11-KT, the key androgen regulating male reproductive function in fish. We also performed histological analyses of the testis and evaluated germ cell accumulation. The ELISA results confirmed that systemic Fsh and Lh levels were higher 1 week after the last rFsh and rLh injections with respect to the control (saline) group (Figures 1A,B). Both rFsh and rLh increased the 11-KT plasma levels, with rLh exerting a more potent stimulatory effect than rFsh (Figure 1C), thus indicating the effective delivery of the recombinant gonadotropins to the testis. The GSI, an indicator of reproductive readiness, increased significantly in the rFsh group with respect to the controls, highlighting its impact on testicular growth. In the rLh-treated males, the GSI was also higher than in the controls, although the differences were not statistically significant (Figure 1D).

Histological examination of germ cell populations revealed different effects of rFsh and rLh on spermatogenesis. The rFsh treatment enhanced the meiosis of germ cells, as indicated by the increased percentage of spermatocytes in the seminiferous tubules of the cortical region of the testis (Figures 1E,F). Conversely, rLh decreased the proportion of spermatids in the cortical and medullar tubules and increased that of spermatozoa in the lumen, indicating a more potent effect of rLh than rFsh at promoting spermiogenesis (Figures 1E–G). The BrdU labelling, as marker for cell proliferation, demonstrated an increased proliferative activity of spermatogonia in the testicular cortical region following rFsh treatment, which was not observed with rLh (Figure 1H). Quantification of BrdU-positive spermatogonia revealed a marked increase in the rFsh group (∼30%) compared to saline (∼8%), whereas rLh did not show a significant effect (∼1% compared to saline) (Figure 1I). These findings suggest that rFsh specifically stimulates the proliferation of early-stage germ cells, potentially augmenting the germ cell pool available for subsequent spermiogenesis enhanced by rLh.

### Transcriptome analysis

**FIGURE 2:**
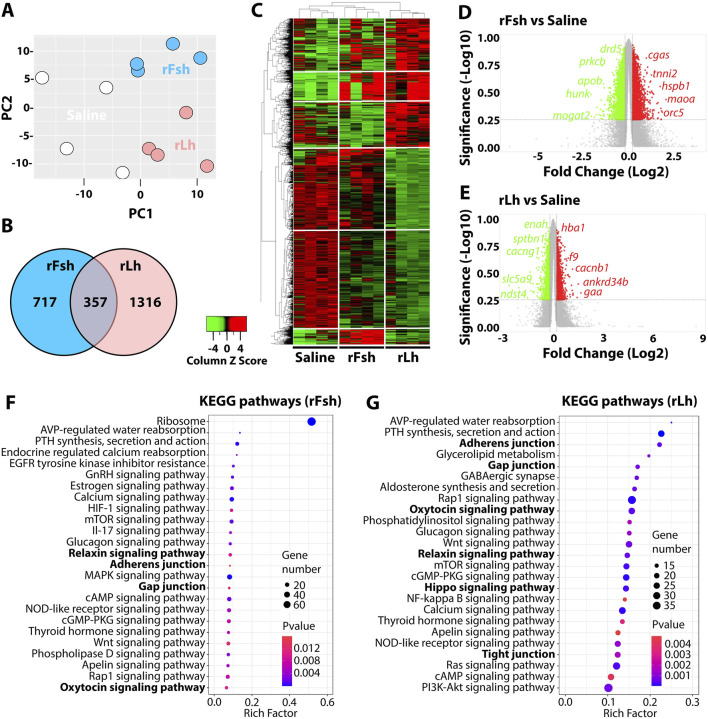
RNA-seq analysis of testis from Senegalese sole males treated with rFsh or Lh. **(A)** Principal component analysis (PCA) of the top 500 differentially expressed genes (DEGs) in testes from males treated with saline (control), rFsh or rLh (n = 4). **(B)** Venn diagrams showing the number of rFsh- and rLh-specific and common DEGs (in intersect region) with respect to the control group. **(C)** Heatmap generated by hierarchical clustering of RNA-seq expression z-scores computed for the 2390 DEGs (*p*-adj <0.01; Log2 fold change >0.5) between treatments. **(D,E)** Volcano plots of DEGs in the rFsh- **(D)** and rLh-treated **(E)** groups with respect to the control group. The x-axis shows Log2 fold changes in expression and the y-axis the negative logarithm of their *p*-value to base 10. Red and green dot mark the genes with significantly increased or decreased expression respectively (FDR <0.01). The most regulated transcripts in each case are indicated. **(F,G)** Canonical pathways related to DEG in the testis of rFsh- **(F)** and rLh-treated **(G)** males identified using the PANTHER classification system. The plot shows the 25 most highly enriched signaling pathways (FDR <0.05) in the testis, with the Rln, Oxt and cell-cell adhesion pathways highlighted in bold lettering. The X-axis shows the rich factor while the Y-axis shows the KEGG pathway. A high and low *P*-value is represented by blue and red, respectively.

A comprehensive RNA-seq analysis was conducted on testis samples from four distinct males per group (saline, rFsh and rLh) to determine DEGs following the hormone treatments. The PCA of the expression data successfully distinguished the treatment groups into three distinct clusters with minimal overlap, indicating that each hormone induced a different transcriptomic profile. This segregation of the data confirmed the robustness of the experimental design and the consistency of the biological replicates (Figure 2A). However, the control group exhibited greater dispersion of the data than the rFsh and rLh groups, reflecting a higher variability in the transcriptomic profile of the fish treated with saline. Out of the 23,179 annotated protein-coding genes, 2,390 DEGs were identified (adjusted *p*-value < 0.01), with 717 genes specific to rFsh, 1,313 specific to rLh, and 357 shared between the two gonadotropins (Supplementary Dataset S1). A Venn diagram further illustrates the distribution of DEGs unique to each treatment and those shared between both treatments (Figure 2B).

The heatmap showing the expression levels of DEGs across the different treatments revealed clusters of genes that were similarly regulated or uniquely modulated by each hormone treatment. Approximately 51% of the DEGs in the rFsh treatment were upregulated, while rLh predominantly downregulated 63% of the DEGs (Figure 2C). Volcano plots for rFsh (Figure 2D) and rLh (Figure 2E) showed the relationship between the extent of gene expression changes (log2 fold change) and statistical significance (*p*-value). Further pathway enrichment analysis confirmed that rFsh and rLh regulate distinct and common biological pathways. For rFsh, the KEGG pathway analysis highlighted specific pathways such as ribosome biogenesis (the most significantly enriched pathway in terms of gene count and *p*-value) and estrogen signaling, which were not influenced by rLh (Figure 2F). In contrast, rLh specifically modulated the GABAergic and Hippo signaling pathways (Figure 2G). Both rFsh and rLh regulated some pathways including Oxt, Rln and thyroid hormone (Tsh) signaling pathways, as well as other pathways related to cell-cell interaction, such as tight junctions, adherens junctions, and gap junctions (Figures 2F,G). To clarify the distinct actions of each hormone, we examined pathways using uniquely regulated genes. These pathways largely overlapped with those identified using all differentially expressed genes. For example, rLh specifically regulates genes involved in adherens, gap, and tight junctions, as well as in the Oxt, Rln, and Hippo signaling pathways (Supplementary Figure S2B). In contrast, rFsh strongly influences the ribosome pathway and also affects GnRH and RLN signaling, but not cell-cell adhesion or OXT signaling - differing from the results using all regulated genes (Supplementary Figure S2A). These findings support that Lh plays a key role in regulating testicular cell-cell adhesion. In line with these results, GO analysis suggested that rFsh predominantly regulates genes with biological functions related to cytoplasmic translation, ribosome assembly or rRNA processing (Supplementary Figure S3A), cellular components such as ribosome, focal adhesion or cell junction (Supplementary Figure S3B), and molecular functions such as translation initiation or ribosome binding (Supplementary Figure S3C). In turn, rLh affects transcripts with predicted biological functions related to mRNA transcription or chromatin organization (Supplementary Figure S3D), cellular components like endoplasmic reticulum or microtubule and cell junction (Supplementary Figure S3E), and molecular functions such as nuclear receptor binding, cadherin binding or transcription coactivator activity (Supplementary Figure S3F).

### Gonadotropic regulation of cell adhesion-related transcripts and proteins

**FIGURE 3:**
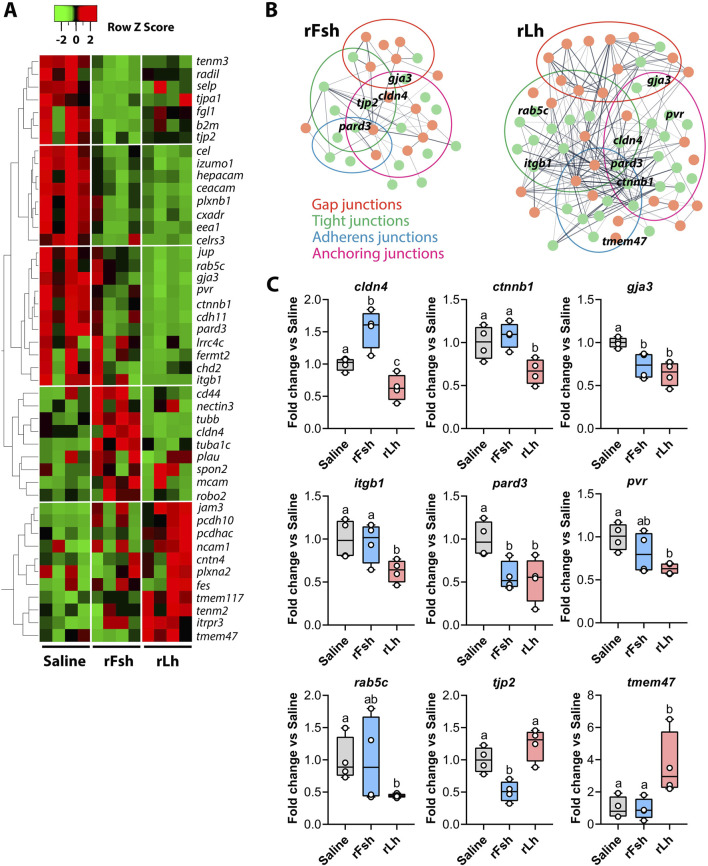
Gonadotropins regulate the expression of cell adhesion molecules. **(A)** Heatmap generated for the DEGs related to cell adhesion molecules (CAMs) as indicated by the PANTHER pathway: gap junctions, adherens junctions and tight junctions. **(B)** Representation of the protein-protein interaction (PPI) networks of DEGs related to CAMs specifically regulated by rFsh (left) or rLh (right). The different colored circles group the DEGs according to the type of junctions where the protein products are potentially located. Representative transcripts from each group are annotated. **(C)** Validation of the RNA-seq data by RT-qPCR for selected genes: claudin 4 (*cldn4*), catenin beta 1 (*ctnnb1*), gap junction protein alpha 3 (*gja3*), integrin subunit beta 1 (*itgb1*), par-3 family cell polarity regulator (*pard3*), poliovirus receptor (*pvr*), RAS oncogene family 5c (*rab5c*), tight junction protein 2 (*tjp2*), and transmembrane protein 42 (*tmem42*). Data are presented as box and whisker plots/scatter dots with horizontal line (inside box) indicating median and outliers (n = 4 fish, white dots), and were statistically analyzed by one-way ANOVA followed by the Tukey’s multiple comparison test. Bars with different superscripts are significantly different (*P* < 0.05).

The heatmap analysis of DEGs potentially associated with cell adhesion processes revealed a predominant downregulation after treatment with the gonadotropins. However, the rLh regulated more than twice the number of genes encoding for potential CAMs compared to rFsh, suggesting a stronger impact of Lh on cell adhesion dynamics within the BTB (Figure 3A). The STRING analysis of protein-protein interactions confirmed that both gonadotropins modulate the expression of transcripts potentially encoding for proteins involved in various cell junctions, including adherens, gap, tight, and anchoring junctions (Figure 3B). Specifically, rFsh downregulated transcripts such as tight junction protein 2 (*tjp2*) and tight junction associated protein 1 (*tjap1*), while rLh selectively downregulated genes including integrin subunit beta 1 (*itgb1*), catenin beta 1 (*ctnnb1*), poliovirus receptor (*pvr*), plakoglobin (*jup*), and claudin 4 (*cldn4*) (Figure 3A). These results were confirmed by RT-qPCR (Figure 3C). Interestingly, *cldn4* was upregulated by rFsh, suggesting differential regulation of this tight junction component in distinct cell types (Figures 3A,C). Additionally, rFsh specifically upregulated tubulin genes (tubulin beta class I, *tubb*, and tubulin alpha 1c, *tuba1c*), whereas rLh upregulated transcripts such as transmembrane proteins 47 and 117 (*tmem47, tmem117*), inositol 1,4,5-trisphosphate receptor type 3 (*itpr3*), and teneurin transmembrane protein 2 (*tenm2*) (Figures 3A,C). The CAMs downregulated by both hormones included gap junction protein alpha 3 (*gja3*) and par-3 family cell polarity regulator (*pard3*) (Figures 3A,C).

**FIGURE 4:**
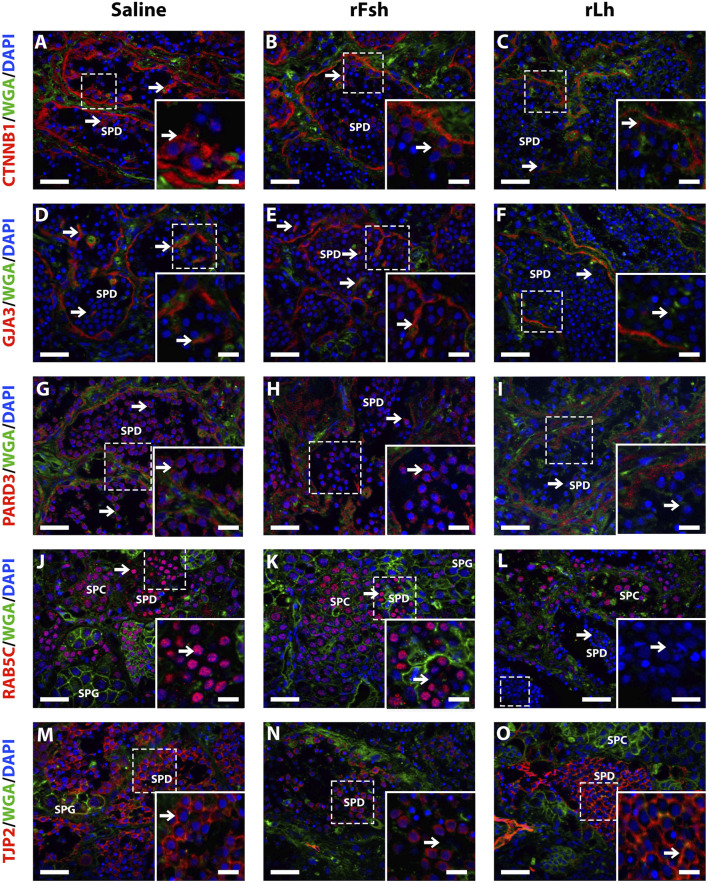
Immunolocalization of Ctnnb1, Gja3, Pard3, Rab5c and Tjp2 in the testis of Senegalese sole treated with saline, rFsh or rLh. The panels show representative immunofluorescence microscopy images of the localization Ctnnb1 **(A–C)**, Gja3 **(D–F)**, Pard3 **(G–I)**, Rab5c **(J–L)** and Tjp2 **(M–O)** in the testis of males treated with saline **(A, D, G, J, M)**, rFsh **(B, E, H, K, N)** or rLh **(C, F, I, L, O)** as indicated. Sections were labelled with commercial rabbit polyclonal antibodies specific for each protein and were visualized with a Cy3-conjugated sheep anti-rabbit (red) antibody. The cell nuclei were counterstained with 4′,6-diamidino-2-phenylindole (DAPI; blue), while the membranes and extracellular matrix were visualized with Alexa Fluor 647-conjugated wheat germ agglutinin (WGA, green). Scale bars, 30 µm. In each panel, the aera that was zoomed-in for the inset is indicated doted lines. The arrows in the insets point the cellular type where the expression of the protein appears to change with the treatment, i.e., Ctnnb1, Gja3 and Tjp2 **(A–F)** and **(M–O)** in Sertoli cells, and for Pard3 and Rab5c **(G–L)** in spermatids. Scale bars, 10 µm (in the inset, 3 µm). SPG, spermatogonia; SPC, spermatocyte; SPD, spermatid; SPZ, spermatozoa.

To determine the specific localization of the regulated cell adhesion and junctional proteins, immunofluorescence microscopy was carried on testicular sections with saline-, rFsh- or rLh-treated males. Commercial antibodies against mammalian proteins with predicted cross-reactivity to sole orthologs based on antigen amino acid alignment were used for immunostaining. Results confirmed the reduced expression of Ctnnb1, Gja3, Pard3 and Tjp2, as well as the regulator of cell-cell junctions Rab5C in response to the gonadotropin treatments (Figure 4). Further detailed immunolocalization revealed that Ctnnb1 and Tjp2 were detected at Sertoli cell-spermatid junctions (Figures 4A,M, insets), while Gja3 and Pard3 were found between Sertoli cells (Figures 4D,G, insets). rLh specifically reduced the protein expression of Rab5c in spermatids (Figures 4J–L, insets), as well as that of Gja3, Pard3 and Ctnnb1 in Sertoli cells projections contacting spermatids (Figures 4A–C, D–F, G–I, insets). In contrast, rFsh downregulated only Ctnnb1 and Tjp2 (Figures 4A–C, M–O, insets).

### Gonadotropins modulate the Oxt signaling pathway

**FIGURE 5:**
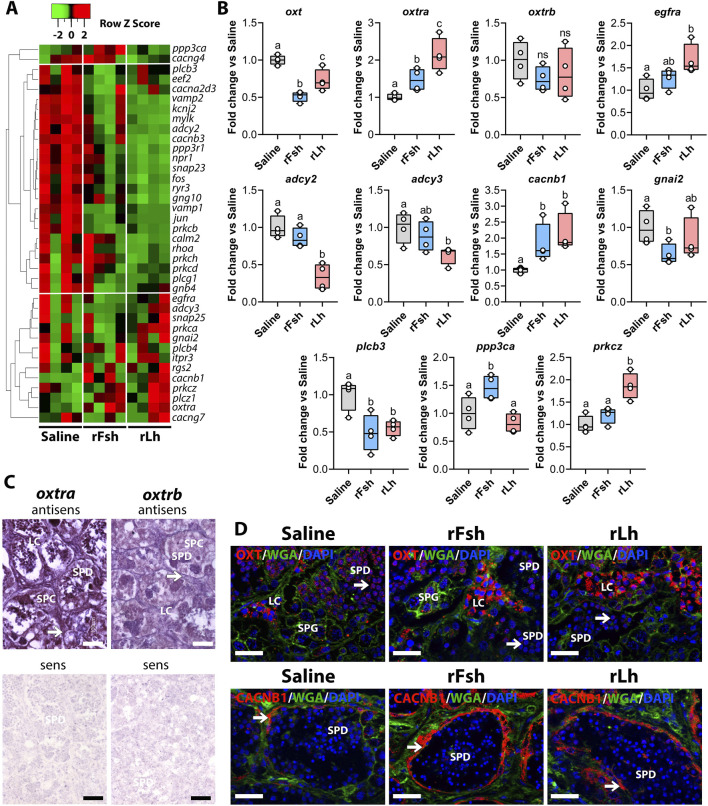
Gonadotropins regulate the oxytocin (Oxt) signalling pathway. **(A)** Heatmap of DEGs related to the Oxt signaling pathway in the rFsh- and rLh-treated groups. **(B)** Validation of the RNA-seq data by RT-qPCR for selected genes potentially involved in the pathway: oxytocin (*oxt*), oxytocin receptor a (*oxtra*), oxytocin receptor b (*oxtrb*), epidermal growth factor receptor a (*egfra*), adenylate cyclase 2 (*adcy2*), adenylate cyclase 3 (*adcy3*), calcium voltage-gated channel auxiliary subunit beta 1 (*cacnb1*), G protein subunit alpha I2 (*gnai2*), phospholipase C beta 3 (*plcb3*), protein phosphatase 3 catalytic subunit alpha (*ppp3ca*), and protein kinase C zeta (*prkcz*). Data are presented as box and whisker plots/scatter dots with horizontal line (inside box) indicating median and outliers (n = 4 fish, white dots), and were statistically analyzed by one-way ANOVA followed by the Tukey’s multiple comparison test. Bars with different superscripts are significantly different (*P* < 0.05). **(C)** Localization of *oxtra* (left) and *oxtrb* (right) transcripts in the sole testis by ISH. Paraffin sections were hybridized with antisense DIG-labeled riboprobes specific for each Oxt receptor (upper panels) as indicated. Control sections (lower panels) were hybridized with sense probes and were negative. Scale bars, 30 μm. **(D)** Immunolocalization of Oxt (upper panel) and Ctnnb1 (lower panel) in the testis of males exposed to different treatments using rabbit polyclonal antibodies specific for each protein (red). The nuclei were counterstained with DAPI (blue), while the membranes and extracellular matrix were visualized with WGA (green). Scale bars, 30 µm. In C and D, arrows point to Sertoli cells. Scale bars, 10 µm. SPG, spermatogonia; SPC, spermatocyte; SPD, spermatid; SPZ, spermatozoa, LC, Leydig cell.

Because the Oxt signaling pathway was regulated by both gonadotropins, we investigated in more detail how rFsh and rLh altered its components. The heatmap analysis of DEGs indicated a more pronounced downregulation of the components of the Oxt signaling pathway by rLh treatment than by rFsh (Figure 5A). Both gonadotropins downregulated jun proto-oncogene, AP-1 transcription factor subunit (*jun*) andphospholipase C beta 3 (*plcb3*), while upregulated genes included calcium voltage-gated channel auxiliary subunit beta 1 (*cacnb1*) and Oxt receptor a (*oxtr*a) (Figure 5A). The rLh treatment specifically downregulated adenylate cyclase 2 (*adcy2)* while it upregulated guanine nucleotide-binding protein G(i) subunit alpha-2 (*gnai2*) and adenylate cyclase 3 (*adcy3*), which also are involved in the Rln signaling pathway (Figure 5A). In contrast, rFsh uniquely upregulated protein phosphatase 3 catalytic subunit alpha (*ppp3ca*) and calmodulin 2 (*calm2*), whereas rLh increased expression of epidermal growth factor receptor a (*egfra*) and protein kinase C zeta (*prkcz*) (Figure 5A). RT-qPCR confirmed RNAseq data for selected genes (*ppp3ca*, *plcb3, adcy2*, *egfra*, *prkcz*, *oxtra*, *oxtrb*, and *cacnb1*. However, disparities appeared for *adcy3* and *gnai2*, since *adcy3* was significantly downtregulated by rLh and *gnai2* by rFsh in the qPCR data. This may be explained by differences in technique sensitivity or the presence of differential isoform expression. Moreover, since *oxtrb* and *oxt* were not detected in the transcriptome, we performed qPCR and successfully identified their trancripts in the testis. *oxt* was downregulated by both gonadotropins, while oxtrb was not changed after hormonal treatment (Figure 5B).

To further explore the functional relevance of these observations, we investigated the spatial distribution of Oxt and its receptors in the testis by ISH using specific riboprobes. The results indicated that both *oxtra* and *oxtrb* were expressed across all testicular cell types, with the highest expression in Leydig cells and spermatids, suggesting that Oxt may exert both paracrine and autocrine effects (Figure 5C). Immunofluorescence microscopy for Oxt revealed strong neuropeptide staining in in Leydig cells and weaker expression in spermatids. Spermatid staining was notably reduced following gonadotropin treatment (Figure 5D). Furthermore, immunostaining confirmed that Cacnb1 localized to Sertoli cells lining the medullar tubules (Figure 5D). Signal intensity increased after rFsh and rLh treatment, in agreement with the mRNA upregulation (Figure 5B).

### Lh regulation of the Hippo signaling pathway

**FIGURE 6:**
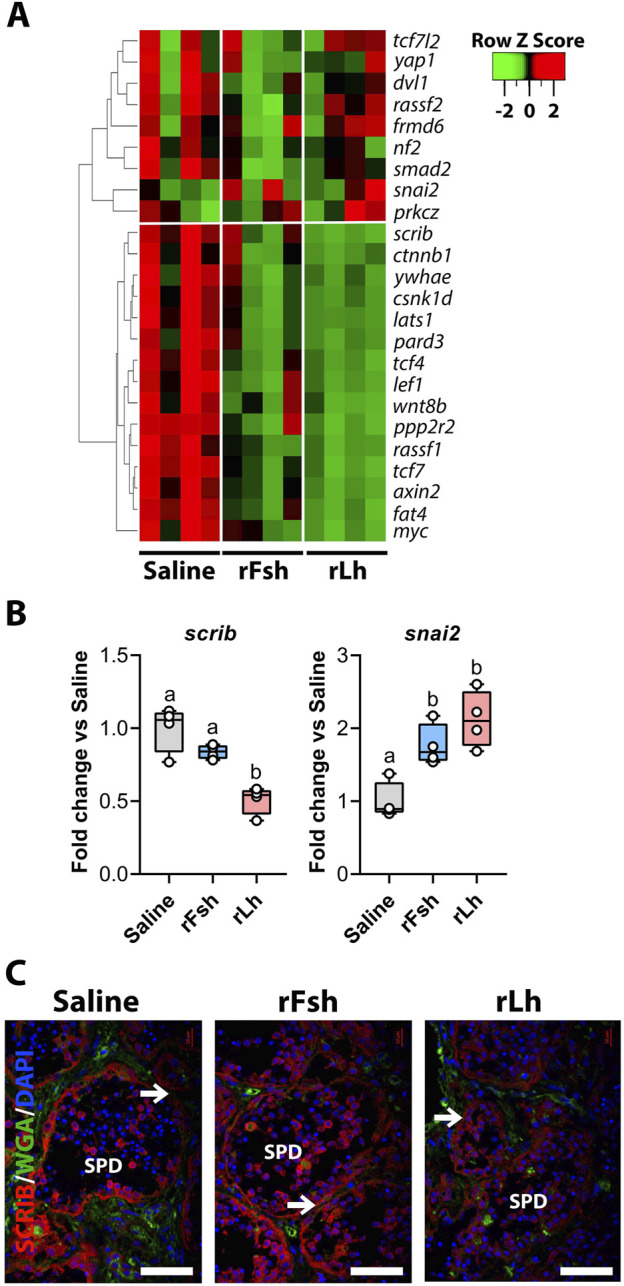
Gonadotropic regulation of the Hippo signaling pathway. **(A)** Heatmap of transcripts related to the Hippo signaling pathway differentially regulated by rFsh and rLh. **(B)** RT-qPCR analysis of transcripts potentially involved in the pathway, such as scribble planar cell polarity protein (*scrib*) and snail family transcriptional repressor 2 (*snai2*). Data are presented as box and whisker plots/scatter dots with horizontal line (inside box) indicating median and outliers (n = 4 fish, white dots), and were statistically analyzed by one-way ANOVA followed by the Tukey’s multiple comparison test. Bars with different superscript are significantly different (*P* < 0.05). **(C)** Immunolocalization of Scrib in the testis of males exposed to different treatments (red), using a rabbit polyclonal antibody specific for SCRIB. The nuclei were counterstained with DAPI (blue), while the membranes and extracellular matrix were visualized with WGA (green). The arrows point to Sertoli cells where the staining appears to be of lower intensity with the rLh treatment. Scale bars, 30 µm. SPD, spermatid.

Since we found that the rLh may specifically regulate the Hippo signaling pathway, which is involved in male sexual maturation, capacitation, and fertilization (Zhang et al., 2019) and in the regulation of BTB cell adhesion (Su et al., 2012; Yu et al., 2015), we further analyzed the transcriptomic data related to this pathway. The heatmap analysis of DEGs potentially involved in the Hippo signaling pathway showed a notable downregulation following rLh treatment, while the rFsh had a minimal impact (Figure 6A). rLh downregulated key Hippo pathway effectors, such as Scribble (*scrib*), large tumor suppressor 1 (*lats1*), and lymphoid enhancer-binding factor 1 (*lef1*), as well as CAMs, such as partitioning defective 3 (*pard3*) and β-catenin (*ctnnb1*) (Figure 6A), suggesting a potential effect of the Hippo signaling pathway in the regulation of cell adhesion. The RT-qPCR experiments corroborated the RNA-seq data for specific genes, including *scrib* and zinc finger protein SNAI2 (*snai2*) (Figure 6B). To localize Scrib, we used a commercial antibody. The protein was expressed in most germ cells and Sertoli cells, and staining was reduced in the medullar region after rLh treatment (Figure 6C).

### Gonadotropin effects on the Rln signaling pathway

**FIGURE 7:**
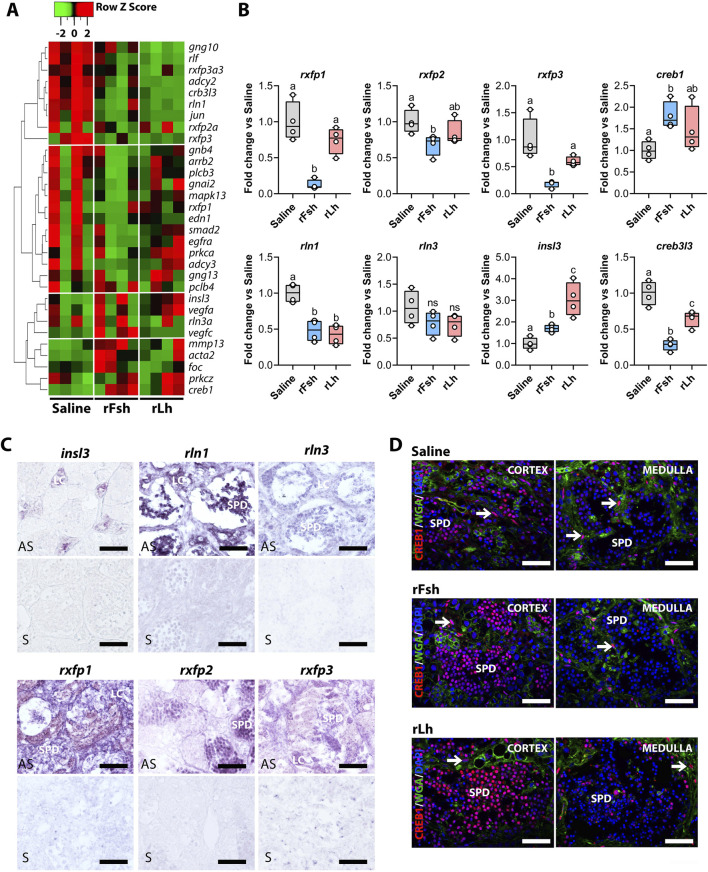
Gonadotropins modulate the Relaxin (Rln) signaling pathway. **(A)** Heatmap of transcripts related to the Rln signaling pathway differentially regulated by rFsh and rLh. **(B)** Validation of the RNA-seq data by RT-qPCR for selected transcripts related to the pathway: relaxin family peptide receptor 1 (*rxfp1*), relaxin family peptide receptor 2 (*rxfp2*), relaxin family peptide receptor 3 (*rxfp3*), cAMP responsive element binding protein 1 (*creb1*), relaxin 1 (*rln1*), relaxin 3 (*rln3*), insulin like 3 (*insl3*), and cAMP responsive element binding protein 3 like 3 (*creb3l3*). Data are presented as box and whisker plots/scatter dots with horizontal line (inside box) indicating median and outliers (n = 4 fish, white dots), and were statistically analyzed by one-way ANOVA followed by the Tukey’s multiple comparison test. Bars with different superscript are significantly different (*P* < 0.05). **(C)** Localization of *insl3*, *rln1*, *rln3* (upper panel) and *rxfp1*, *rxfp2* and *rxfp3* (lower panel) transcripts in the sole testis by ISH with antisense (AS) DIG-labelled riboprobes specific for each transcript as indicated. Control sections hybridized with sense probes (S) were negative. Scale bars, 30 μm. **(D)** Immunolocalization of Creb1 in the testis of males exposed to different treatments in the cortex (left) and medullar (right) regions of the testis (red). The nuclei and membranes were counterstained with DAPI (blue) and WGA (green), respectively. Arrows indicate the nuclei of Sertoli cells. Scale bars, 30 µm. LC, Leydig cells; SPD, spermatids.

DEGs in the Rln signaling pathway are differentially regulated by rFsh and rLh, but no consistent pattern of global up- or downregulation emerged (Figure 7A). The RT-qPCR data revealed however that insulin-like 3 (*insl3*) mRNA was upregulated by both rFsh and rLh, while the relaxin 1 (*rln1*) and relaxin 3 (*rln3*) transcripts were downregulated by both gonadotropins (Figure 7B). Interestingly, the expression of the three Rln receptors (*rxfp1*, *rxfp2*, and *rxfp3*) was exclusively downregulated by rFsh but remained unaffected by rLh. Additionally, the mRNAs encoding downstream effectors of the Rln signaling pathway, such as the cAMP responsive element-binding protein 3-like 3 (*creb3l3*) and cAMP responsive element-binding protein 1 (*creb1*), showed differential gonadotropic regulation. Both rFsh and rLh downregulated c*reb3l3* and upregulated *creb1*, although only statistically significant for rFsh (Figure 7B). ISH showed that *insl3* was restricted to Leydig cells, while both *rln1* and *rln3* were detected in spermatids and Leydig cells (Figure 7C). The Rln receptors *rxfp1* and *rxfp3* were expressed in most testicular cell types, whereas *rxfp2* was specific to spermatids (Figure 7C). All control sense probes yielded negative results.

Finally, to validate the regulation of the downstream effectors of the Rln signaling pathway, we conducted immunofluorescence for Creb1. It was detected in the nucleus of immature spermatids from the cortical region and weaker in the medullar spermatids (Figure 7D). Both gonadotropins increased Creb1 staining in cortical immature spermatids, but not in the spermatids from the medulla (Figure 7D), in agreement with the RT-qPCR data.

### *In vitro* effects of gonadotropins, Oxt and Rln on the testicular expression of cell junction related transcripts

**FIGURE 8:**
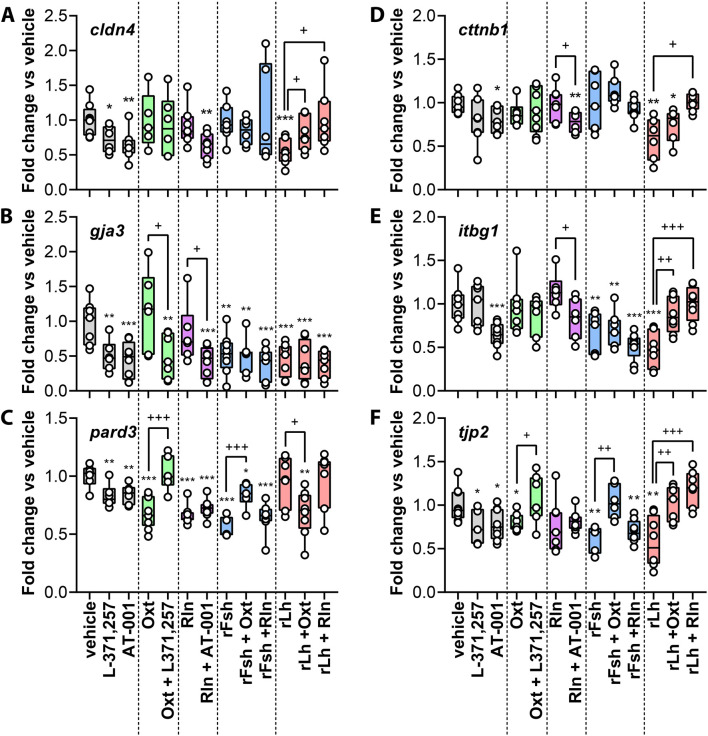
Effect of gonadotropins, Rln and Oxt, and pharmacological inhibition of their receptors, on the expression of testicular transcripts encoding CAMs *in vitro*. **(A–F)** Testicular explants were treated with 100 ng/mL rFsh, rLh Rln or Oxt, or with a combination of rFsh or rLh with Rln or Oxt, for 24 h. The negative control was only incubated with the vehicle (0.1% DMSO). Additional explants treated with vehicle, Rln or Oxt were incubated with 10 µM of Rln and Oxt receptor antagonists (AT-001 and L-371,257, respectively) for 1 h prior to stimulation with the corresponding peptide hormone. The expression of selected transcripts encoding for CAMs, indicated in each panel, was subsequently carried out by RT-qPCR. The values represent compiled data from three independent experiments using explants from different males, each with two replicates per treatment. The data are presented as box and whisker plots/scatter dots with horizontal line (inside box) indicating median and outliers (n = 4 fish, white dots), and were statistically analyzed by the two-tailed unpaired Student’s t-test, or by the nonparametric Mann Whitney test when variances were not equal. *, *P* < 0.05; **, *P* < 0.01; ***, *P* < 0.001, with respect to the control; ^+^, *P* < 0.05; ^++^, *P* < 0.01, ^+++^, *P* < 0.001, between groups as indicated by brackets.

To explore how various hormones directly affect the expression of cell adhesion-related transcripts, we carried out *in vitro* experiments using testicular explants followed by RT-qPCR analysis (Figure 8). The explants were treated for 24 h with rFsh, rLh, Oxt, or Rln, and combinations of both gonadotropins with Oxt or Rln. Specific inhibitors of Oxt receptors (L-371,257) and Rln receptors (AT-001) were also employed to validate the involvement of the Oxt and Rln signaling pathways. qRT-PCR showed that 24-h exposure to rLh significantly *cldn4*, *ctnnb1*, *gja3*, *itgb1* and *tjp2*, but not *pard3* (Figure 8). The rFsh similarly inhibited the expression of *gja3*, *pard3*, *tjp2*, and *itgb1*, but did not affect that of *cldn4* or *ctnnb1* (Figures 8A–F). These data align with the RNA-seq data after 6 weeks of exposure to the hormones *in vivo*. Despite RNA-seq data indicated the gonadotropic regulation of the Rln and Oxt signaling pathways, direct exposure of the explants with Rln or Oxt had minimal effect except for *pard3* (Figure 8C), which was reduced by both peptides, and *tjp2* (Figure 8F), which was significantly downregulated by Oxt. For both *pard3* and *tjp2*, the negative effect of Oxt was reversed with L-371,257, while that of Rln on *pard3* could not be prevented with AT-001, suggesting that in this case the Rln receptor involved may not be sensitive to the inhibitor. The absence of regulation of *cldn4*, *cttnb1*, *gja3* and *itgb1* by Oxt and Rln *in vitro* (Figures 8A,B,D,E) suggest that longer exposure to the peptides may be required for regulatory effects. Nevertheless, despite treatment, Oxt and Rln receptor inhibitors showed a general reduction in the expression of the cell adhesion-related transcripts, though *cttnb1* and *itgb1* were only reduced by AT-001. The data also indicated that when rFsh was combined with Oxt, the neuropeptide counteracted the downregulation of *tjp2* and *pard3*, while Rln had no such effect (Figure 8). Conversely, the rFsh-mediated downregulation of *cldn4*, *ctnnb1, gja3* and *itgb1* was not reversed by Oxt or Rln. Under rLh treatment, the downregulation of *cldn4*, *itgb1*, *ctnnb1*, and *tjp2* expression was recovered by both Oxt and Rln, whereas that of *gja3* was not restored by either of the peptides (Figures 8A,B,D,E). Finally, *pard3* expression was inhibited by rLh plus Oxt but unaffected by rLh and Rln (Figure 8C).

In summary, the gonadotropic inhibition of CAMs expression is mediated by the impairement of the Oxt and Rln signaling pathways. However, gonadotropins can also regulate the expression of some gap junction genes (*gja3*) through Rln- and Oxt-independent pathways.

## Discussion

By integrating histological, transcriptomic and immunological approaches, the present study provides a comprehensive analysis of the effects of Fsh and Lh on spermatogenesis in a flatfish with semi-cystic type of germ cell development. Our data further highlight the critical role of the gonadotropins in the regulation of cell adhesion and junctional processes during spermiogenesis and spermiation. These effects occur through the differential modulation of the Oxt and Rln signaling pathways, as well as through independent processes.

In teleosts, spermatogenesis is regulated by the interaction of gonadotropins with local factors (Schulz et al., 2023). In the Senegalese sole, the Fsh stimulates early-stage germ cell proliferation, enhancing testicular growth and spermatocyte development, while the Lh predominantly influences later stages of spermatogenesis by promoting haploid germ cell maturation and spermatozoa differentiation (Chauvigné et al., 2017; Chauvigné et al., 2022; Schulz et al., 2023). Both Fsh and Lh however play steroidogenic roles by activating the synthesis and secretion of the androgen 11-KT by Leydig cells (Chauvigné et al., 2012). Our transcriptomic analysis reveals that Fsh and Lh regulate both overlapping and unique molecular pathways in the sole testis. The data indicate that Fsh downregulates genes related to ribosome biogenesis, likely linked to meiosis stimulation through translational elongation suppression (Sabi and Tuller, 2019), and affects the estrogen signaling pathway, promoting the growth of type A undifferentiated spermatogonia (Kobayashi et al., 2011; de Castro Assis et al., 2018). In contrast, Lh primarily modulates the GABAergic system, an inhibitory regulator of spermatogonial stem cell division, which may also influence spermiogenesis (Kanbara et al., 2005; Du et al., 2013).

The transcripts regulated by Fsh and Lh, especially those involved in testicular cell adhesion and communication pathways at the BTB and Sertoli cell-spermatid junctions, underscores the role of gonadotropins in the remodelling of the sole testis. The adherens junction protein CTNNB1 and integrin signaling component ITGB1, important for cell growth and adhesion, regulate mammalian spermatogonial stem cell proliferation, and are crucial for spermatogenesis and the integrity and restructuring of the BTB (de Freitas et al., 2016; Sá et al., 2013; Wang et al., 2019). In the sole testis, both *ctnnb1* and *itgb1* transcripts were specifically downregulated by Lh through the Rln pathway. Downregulation of *itgb1* by rLh likely results from the androgenic activity of Leydig cells (Wang et al., 2019). The cytoskeletal regulator Scrib was also downregulated by Lh, indicating a possible additional effect of the hormone on cellular polarity during spermiation. Finally, the *rab5c*, whose protein product of which is involved in intracellular trafficking and fusion at adherens junctions during mammalian spermiogenesis (Lin et al., 2017; Inagaki et al., 2006; Adams and Wayne Vogl, 2017), was downregulated by rLh but unaffected by rFsh, suggesting that Lh specifically influences the integrity of the adherens junctions in the sole testis.

The component of tight junctions Tjp2, which contributes to BTB integrity, was localized in sole Sertoli cells surrounding immature spermatids and was downregulated by rFsh, thus potentially compromising BTB integrity similar to observations in mice (Fink et al., 2006; Xu et al., 2009; Chakraborty et al., 2014). In contrast, the epithelial tight junction protein *cldn4*, involved in paracellular permeability, was upregulated by rFsh and downregulated by rLh, indicating opposing effects of gonadotropins in the transcriptional regulation *cldn4*, in a similar fashion to that reported for some steroidogenic enzymes in sole Leydig cells (Chauvigné et al., 2014b). In rodents, when spermatogenesis is active under FSH stimulation, a relocalization of claudins occurs in the BTB, which tightens its permeability (Tarulli et al., 2006; McCabe et al., 2010). Therefore, it is possible that in sole Fsh regulates Cldn4 protein expression and localization at the level of the BTB in the cortical seminiferous tubules containing migrating spermatocytes, as in rodents (Chihara et al., 2013). Conversely, Lh may induce the leaking of BTB permeability through the downregulation of *cldn4* and specifically be involved in spermatid release in the medullar tubules. However, since we were unable to find a commercial antibody cross-reacting with sole Cldn4, this hypothesis should be tested in future studies. Other proteins interacting with tight junctions were also differentially regulated by gonadotropins. The adapter protein *pard3*, involved in polarized growth and asymmetric cell division (Wong et al., 2008b), was downregulated by both Fsh and Lh, particularly in spermatids, indicating that gonadotropins may prevent tight junction assembly. In turn, the junctional adhesion protein-encoding transcript *jam3*, the protein product of which mediates Sertoli cell-spermatid adhesion (Fujita et al., 2007), was upregulated by rLh, possibly compensating for the loss of *pard3* (Ebnet et al., 2003). Both gonadotropins also affected the expression of gap junction genes in the testis, with decreased mRNA and protein expression of Gja3 (Cx46) in Sertoli cell digitations which is associated with germ cell detachment and apoptosis due to BTB disruption (Pelletier et al., 2015). However, whether the effects of Fsh on the modulation of tight and gap junctions between Sertoli cells and spermatids are mediated directly through the Fsh receptor on Sertoli cells (Chauvigné et al., 2012), or indirectly through androgen pathways as seen in mammals (Lui and Cheng, 2012), remains unclear. In addition, the direct effect of Lh on the expression of junctional genes in spermatids is unlikely, since Lhcgrba is expressed on these haploid germ cells only after their release into the seminiferous lumen (Chauvigné et al., 2014a).

The treatment of sole males with gonadotropins, particularly with rLh, may also regulate testicular cell adhesion through the Hippo pathway, which in mammals governs cell polarity and cell-to-cell adhesion in response to FSH (Sen Sharma et al., 2019). Most of Hippo pathway components were downregulated by rLh. This suggests that this hormone facilitates spermatid detachment and promotes spermiogenesis by modulating cell junction integrity (Su et al., 2012; Karaman and Halder, 2018). In contrast, the rFsh downregulates the testicular expression of some of the pathway components involved in cell contact inhibition, such as the transcriptional coactivator YAP1 (*yap1*) and the Dishevelled-1 cytoplasmic protein (*dvl1*) (Lee et al., 2018; Noce et al., 2019; Ma et al., 2006). The sole ortholog of the Hippo effector SCRIB (*scrib*), crucial for Sertoli cell-spermatid adhesion and BTB integrity (Su et al., 2012), was strongly downregulated by rLh and not affected by rFsh. Both Fsh and Lh downregulated *lats1*, a component of the Hippo pathway affecting cell junctions (Abou Nader et al., 2024). Conversely, *snai2*, a transcriptional repressor of occludins (Micati et al., 2020), was upregulated. These changes reinforce the idea that both gonadotropins repress cell adhesion. Altogether, our results show that gonadotropins fine-tune the Hippo signaling pathway to regulate spermatogenesis and cell junction dynamics and the seminiferous tubule integrity.

The *in vitro* experiments revealed that the gonadotropic regulation of CAMs in the sole testis was in most cases mediated by the Oxt and Rln signaling pathways. The RLN signaling pathway involves several RLN-like peptides (INSL3, RLN1, RLN2, RLN3), which interact with their respective receptors (RXFP1, RXFP2, RXFP3). The INSL3 is crucial for testicular function, influencing germ cell development, Leydig cell activity, and testosterone production through both autocrine and paracrine mechanisms (Ivell et al., 2013; 2020). We observed that *insl3*, *rln1* and *rln3a*, as well as the Rln receptors *rxfp1*, *rxfp2* and *rxfp3*, are expressed in the sole testis. In contrast, the orthologue of mammalian RLN2 (*rln3b* in teleosts) was not detected in testis either in the RNAseq data or by qPCR, which is consistent with the species-specific paralog distribution of Rln peptide in teleosts (Good-Avila et al., 2009).

In mammals, the RLN1 produced by Leydig and Sertoli cells acts through the RXFP1 to regulate Sertoli cell proliferation and seminiferous tubule structure (Edsgärd et al., 2011; Pimenta et al., 2015). In sole, the presence of *rxfp1* expression in sole Sertoli cells and spermatids suggest that the Rln signaling pathway influences both early and late stages of germ cell development. In the zebrafish (*Danio rerio*), the Insl3 signaling enhances spermatogonial differentiation via the Rxfp2 in Sertoli cells (Crespo et al., 2021), while in sole the expression of *rxfp2* in spermatids suggests a role of Insl3 during spermiogenesis and/or spermiation. Also, the Rln3 seems to play a significant role during Nile tilapia (*Oreochromis niloticus*) spermatogenesis, since mutations in *rln3a* and *rln3b* result in male infertility (Yang et al., 2020; Xu et al., 2024). In the Senegalese sole, both *rln3* and *rxfp3* are primarily expressed in spermatids, with lower levels in Leydig cells. *rxfp3* was also present in Sertoli cells. In summary, the cell localization pattern of Rln transcripts in the sole testicular somatic and germ cells resembles to that reported in mammals (Heidari et al., 2018; Pitia et al., 2015), suggesting that these peptide hormones may also regulate fish spermatogenesis via autocrine and paracrine mechanisms.

In our experiments, both gonadotropins downregulate *rln1*, while only Fsh had a strong effect on all three receptors. In mammals, RLN3-RXFP3 interaction activates downstream casacades involving cAMP-producing enzymes (e.g., ADCY), MAP kinases, and transcription factors such as NF-kB and AP-1 (Kocan et al., 2016; van der Westhuizen et al., 2007). This pathway promotes germ cell differentiation, spermatid maturation and androgen production (Baburski et al., 2017). Consistently, we observed that in sole the expression of *adcy2* is downregulated by rLh treatment, which may disrupt the BTB and impair spermatogenesis as observed in mice (Chen et al., 2022). In sole, gonadotropin treatment also influenced transcription factors from the Rln signaling pathway, such as *jun* and *creb1.* These factors are the crucial in the transcriptional regulation of nectin-2, an adherens junction protein essential for Sertoli-germ cells attachment (Lui et al., 2006). Altogether, our findings support the notion that the Rln signaling pathway may regulate cell adhesion in the sole testis through gonadotropin action. This was supported by our *in vitro* study, where the Rln receptor inhibitor AT-001 downregulates all studied CAMs, regardless of Rln presence, mimicking the effect of rLh and/or rFsh. However, when gonadotropin treatment was combined with Rln, only the downregulatory effect of Lh was rescued. This suggests that dysregulation of CAM gene transcription by Lh is mostly dependent on Rln, while that induced by Fsh may occur through the Oxt signaling pathway.

OXT, synthesized by Leydig cells, regulates spermatogenesis and acting on OXTR in Sertoli cells. This signaling modulates seminiferous tubule contractility (Stadler et al., 2020; Frayne et al., 1996). In fish, the role of Oxt has been less explored, although mRNAs for both the Oxt ligand and its receptors (*oxtra* and *oxtrb*) have been identified in the testis of various fish species (Mennigen et al., 2022). In the Senegalese sole, Oxt and *oxtra* and *oxtrb* were detected in spermatids, suggesting an autocrine effect of the neuropeptide in these cells. The OXTR is a G-protein-coupled receptor that activates calcium (Ca^2+^)-dependent signaling pathways, including protein kinase C alpha type (PRKCA), without influencing androgen, estrogen, or progestin synthesis (Mennigen et al., 2022). The effect of OXT is then different to that of the closely related peptide Arginine Vasotocin (AVT), that was recently shown to stimulate spermiogenesis through an androgen-dependent mechanism (Zanardini et al., 2024). The Ca^2+^ influx induced by the binding of OXT to the OXTR is crucial for seminiferous tubule contraction and possibly spermatid release (Fleck et al., 2021). In sole, gonadotropins regulated several Ca^2+^ signaling components. *cacnb1* was upregulated by rFsh and rLh *while cacng4* was downregulated by rLh, and *cacna2d3* and *cacnb3* were downregulated by both gonadotropins. Other genes potentially involved in seminiferous tubule contraction necessary for sperm release and transport, such as *rhoa* and *mylk*, or involved in calcium dynamics under OXT stimulation and critical for spermatid development, such as *plcb3,* were also regulated by rFsh and rLh. Altogether, these results suggest that Oxt mediates the integrity of the seminiferous tubules in the sole testis. This was confirmed *in vitro* where Oxtr inhibitor L-371,257 inhibited the expression of most of the CAMs studied. However, the interaction between the gonadotropins and Oxt revealed that both rFsh and rLh downregulated *tjp2* expression through Oxt, while that of *pard3* was dependent on Oxt only under rFsh treatment. In contrast, the gonadotropic downregulation of *ctnnb1* or *itgb1* was independent of Oxt under rFsh exposure, as opposed to rLh. Finally, the expression of *gja3* was strongly inhibited by gonadotropins, but independently of the Oxt signaling. Therefore, these data suggest that Fsh and Lh disrupt cell-cell junction integrity in the sole testis through different pathways.

**FIGURE 9:**
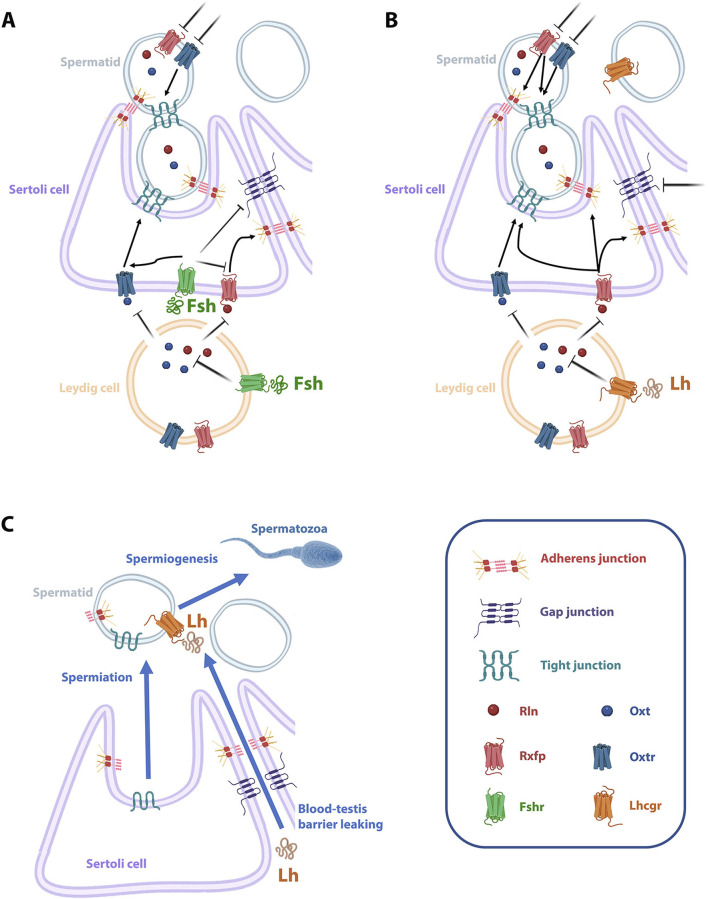
Proposed model of the signaling pathways regulating CAMs during Senegalese sole spermiogenesis and spermiation. **(A)** The activation of the Fsh cognate receptor Fshr by Fsh in Leydig cells downregulates the Oxt and Rln synthesis, which induces the inhibition of the Oxt/Oxtr signaling pathway in Sertoli cells, thereby decreasing the synthesis of tight junction proteins between spermatids and Sertoli cells. Also, through Fshra activation in the Sertoli cells, Fsh directly inhibits the expression of gap junction proteins conforming the BTB (interaction between adjacent Sertoli cells). **(B)** Lh also reduces both Rln and Oxt synthesis in Leydig cells through the activation of the Lhcg. Thus, both Oxtr and Rxfp signaling pathways are inhibited in Sertoli cells, which impedes the proper expression of junctional proteins between Sertoli cells and between Sertoli cells and immature spermatids. **(C)** As a result, gonadotropins facilitate the release of spermatids from Sertoli cells and the leaking of the BTB, which allow macromolecules such as Lh to reach the lumen of the seminiferous tubule and activate the released Lhcgr-expressing mature spermatids to induce spermatozoa differentiation. The figure was drawn using Biorender (https://www.biorender.com).

Based on our findings, we propose a model illustrating how gonadotropins regulate junctional proteins in the Senegalese sole testis (Figure 9). The activation of the Fshra by Fsh in Leydig cells leads to the downregulation of Oxt and Rln synthesis, which inhibits the Oxt/Oxtr signaling pathway in Sertoli cells and impairs the production of tight junction proteins between Sertoli cells and immature spermatids (Figure 9A). In addition, Fsh directly suppresses the expression of gap junction proteins forming the BTB between Sertoli cells, while the adherens junctions may be regulated indirectly by Fsh through the downregulation of the Rxfp in Sertoli cells (Figure 9A). Lh similarly reduces Rln and Oxt synthesis in Leydig cells through activation of Lhcgr, leading to the inhibition of both the Oxtr and Rxfp signaling pathways in Sertoli cells and the disruption of the BTB, as well as the proper expression of adherens and tight junction proteins between Sertoli cells and spermatids (Figure 9B). However, Lh can regulate the gap junctions at the BTB by an independent mechanism (Figure 9B). As a result, gonadotropins facilitate spermatid release from Sertoli cells and the leakage of the BTB (Figure 9C). Thus, macromolecules such as Lh can reach the seminiferous lumen and stimulate the differentiation of Lhcgr-expressing mature spermatids to spermatozoa.

## Conclusion

In summary, this study emphasizes the essential roles of gonadotropins in regulating spermatogenesis in Senegalese sole through the modulation of cell adhesion processes, primarily by the downregulating of the OXT and RLN signaling pathways. Our findings suggest that Fsh appears to act mainly through the Oxt pathway, while Lh seems to inhibit both pathways. However, some junctional proteins appear to be regulated by the gonadotropins through Oxt and Rln independent mechanisms. The Hippo pathway complements these systems maintaining the structural dynamics of the seminiferous tubules necessary to support and nourish germ cell development. These findings underline the intricate interplay between gonadotropin signaling, local paracrine and autocrine hormonal pathways, and the regulation of adhesion molecules, during sole testicular spermatogenesis. Future research is however needed to elucidate the interactions between these mechanisms and gain insight into the understanding of the testicular homeostasis and reproductive health in fish.

## Data Availability

The datasets presented in this study can be found in online repositories. The names of the repository/repositories and accession number(s) can be found below: https://www.ncbi.nlm.nih.gov/geo/, GSE279689.

## Supplementary material

SUPPLEMENTARY FIGURE S1
Correlation between RNAseq results (X) and qPCR results (Y). The z-score for the mean of the results from both techniques were plotted for each treatment group. The coefficient of correlation was of 0.83; 0.69 and 0.70 for Saline, rFsh and rLh, respectively, indicating more variability in the hormone-treated groups despite RNAseq data were mostly confirmed by qPCR.

SUPPLEMENTARY FIGURE S2
Canonical pathways related to unique DEG in the testis of rFsh- **(A)** and rLh-treated **(B)** males identified using the PANTHER classification system. The plot shows the 25 most highly enriched signaling pathways (FDR <0.05) in the testis. The X-axis shows the rich factor while the Y-axis shows the KEGG pathway. A high and low *P*-value is represented by blue and red, respectively.

SUPPLEMENTARY FIGURE S3
Gene ontology (GO) analysis of differentially expressed genes (DEGs) in the Senegalese sole testes of males treated with rFsh or rLh. DEGs were classified according to the GO terms biological process **(A,D)**, cellular component **(B,E)** and molecular function **(C,F)** for treatment with rFsh **(A–C)** or rLh **(D–F)**. For each GO term, the number of differentially expressed transcripts detected in the RNA-seq is indicated by the size of the circle, while the P-value is represented with a color scale. The X-axis represents the enrichment factor.
