# Supplementary figures and images for: Gonadotropins differentially regulate testicular cell adhesion and junctional complexes during flatfish spermiogenesis through the oxytocin and relaxin signaling pathways
Source: Front Cell Dev Biol. 2025 Jun 2;13:1574690. doi: 10.3389/fcell.2025.1574690 (PMC12171224; doi:10.3389/fcell.2025.1574690)

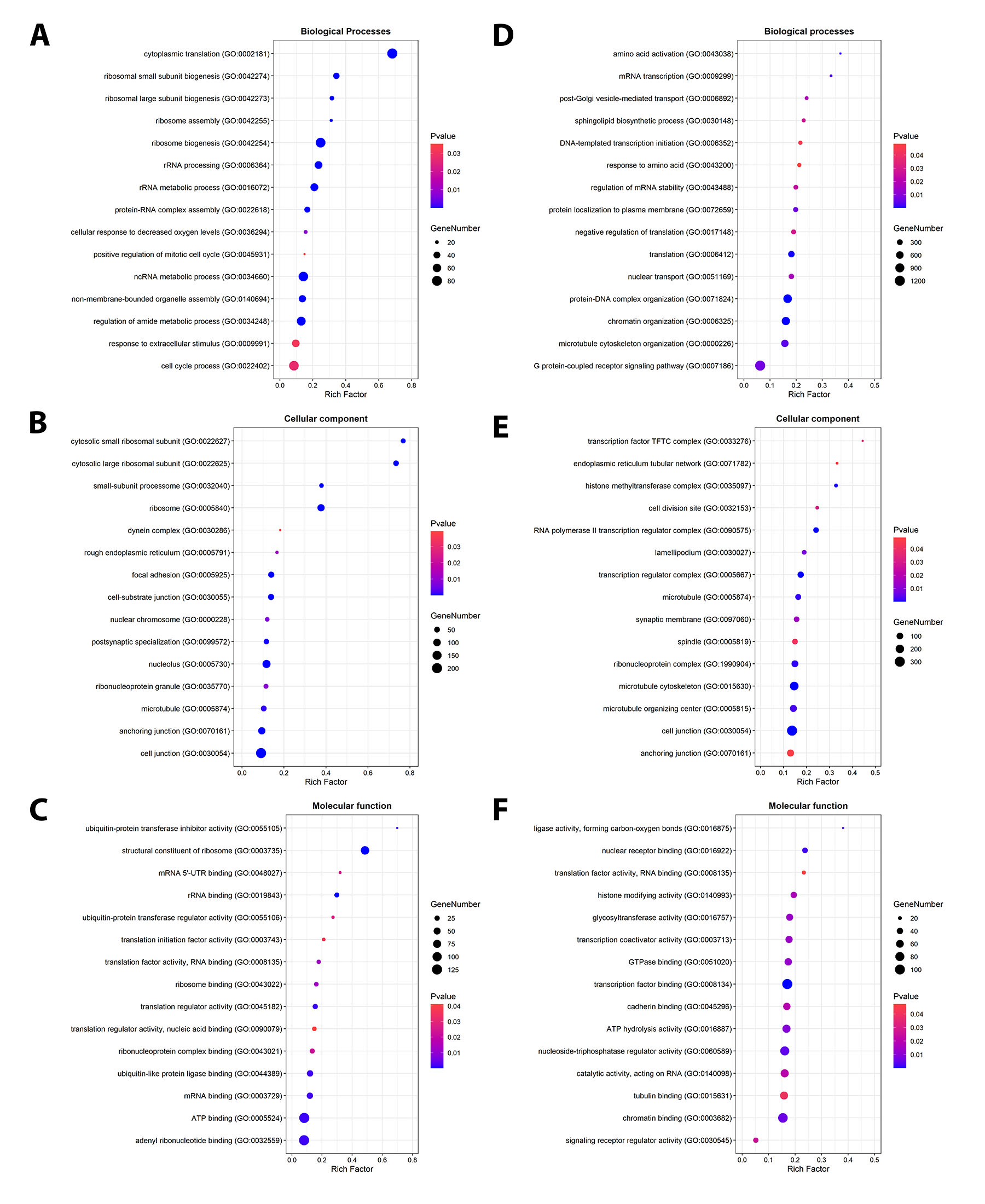

Supplement: Supplementary file 1 [file Image3.jpeg]

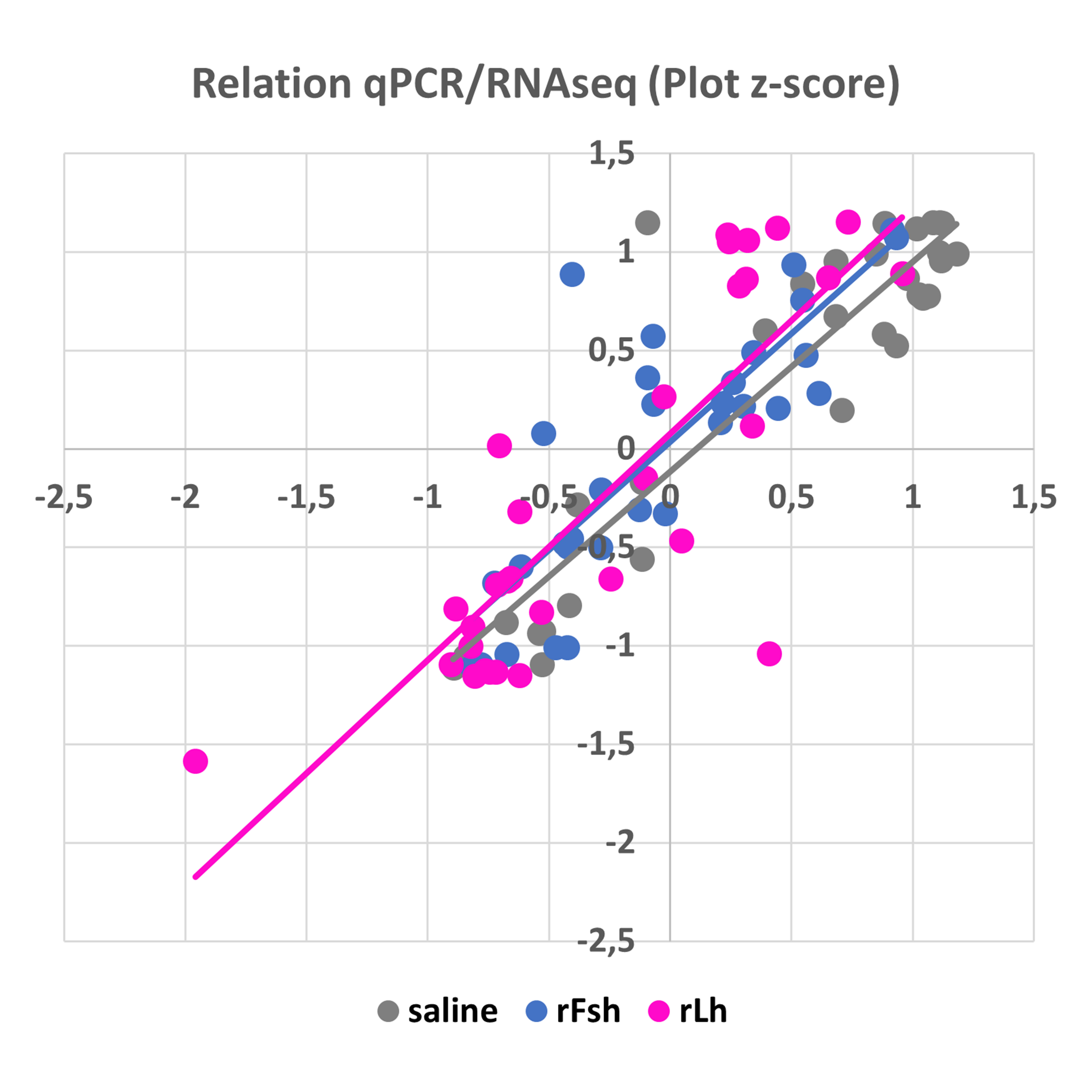

Supplement: Supplementary file 4 [file Image1.jpeg]

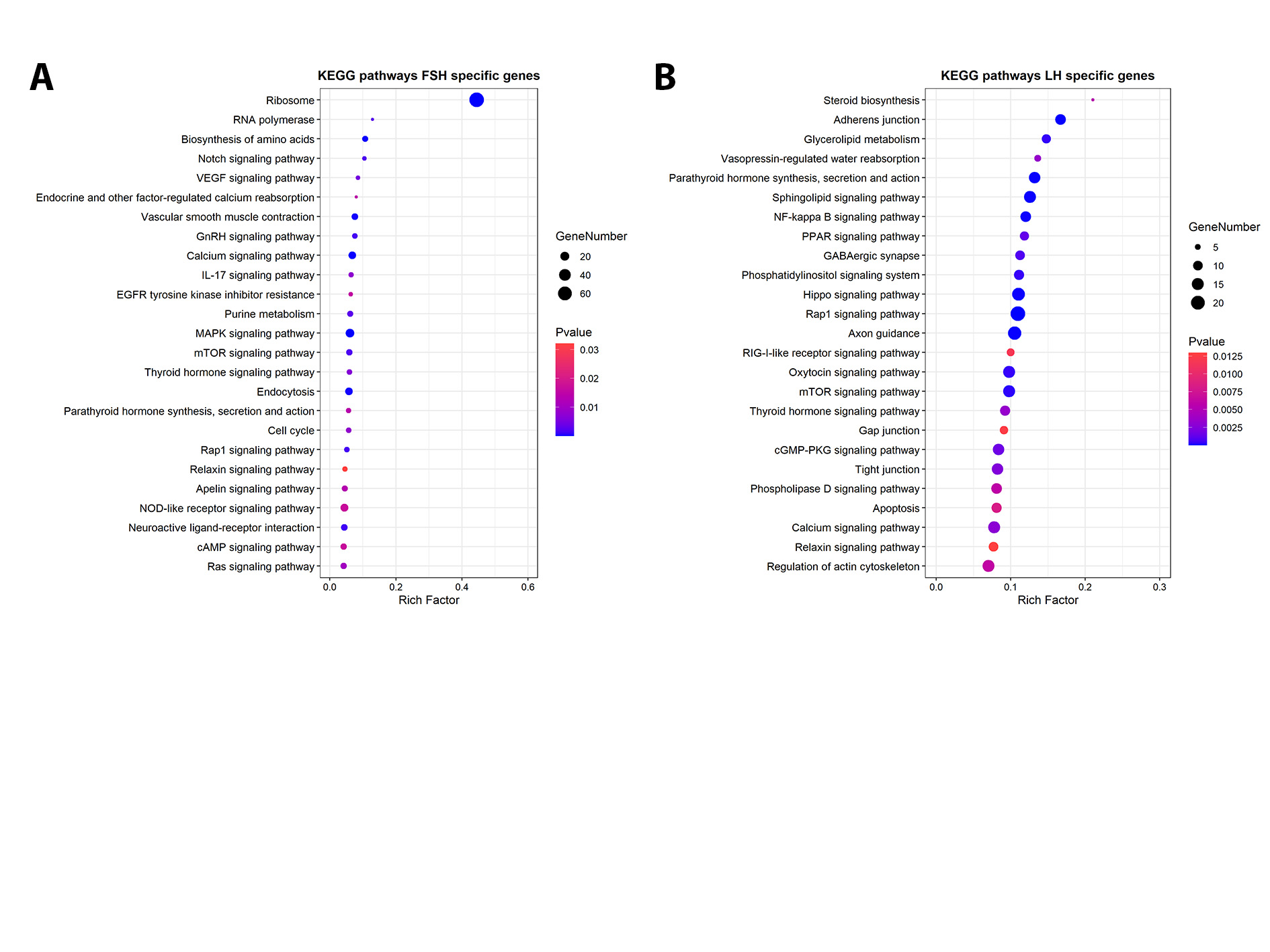

Supplement: Supplementary file 5 [file Image2.jpeg]
