# Supplementary material for: Gonadotropins differentially regulate testicular cell adhesion and junctional complexes during flatfish spermiogenesis through the oxytocin and relaxin signaling pathways
Source: Front Cell Dev Biol. 2025 Jun 2;13:1574690. doi: 10.3389/fcell.2025.1574690 (PMC12171224; doi:10.3389/fcell.2025.1574690)
Supplement: Supplementary file 3 [file Table1.docx]

**TABLE S1**

Oligonucleotide sequences used for qRT-PCR. All primers are flanking one intron for each gene.

| **Name** | **DNA sequence** | **Direction** |
| --- | --- | --- |
| ADCY2-F | 5’-AGCAGCATCCACTGATTGTG-3’ | Forward |
| ADCY2-R | 5’-CTCGATGCAGGCAAGTACAA-3’ | Reverse |
| ADCY3-F | 5’-ACCCTGGTTTTAGGGGTGAC-3’ | Forward |
| ADCY3-R | 5’-GTACTTCCTGTCCGCCATGT-3’ | Reverse |
| CACNB1-F | 5’-TGCGGACAAATGTCGGTTAT-3’ | Forward |
| CACNB1-R | 5’-ACCTCACAGCCTTCCTTCAC-3’ | Reverse |
| CLDN4_F | 5’-CAGCATCCTCAGTGTCCTCAT-3’ | Forward |
| CLDN4_R | 5’-GACGGGGATGATCACGAG-3’ | Reverse |
| CREB1-F | 5’-CGCATGCAATTACCACAGTT-3’ | Forward |
| CREB1-R | 5’-AGCTGCTGCATTAGTCATGG-3’ | Reverse |
| CREB3L3L-F | 5’-GATCTGTTGGGGATGCTGTT-3’ | Forward |
| CREB3L3L-R | 5’-AGTCCCCAAAGTGTCCTCCT-3’ | Reverse |
| CTNNB1_F | 5’-GTGCAATCCCAGAGCTAACC-3’ | Forward |
| CTNNB1_R | 5’-CGATGCCTCCTTCTTTGACA-3’ | Reverse |
| EGFRA-F | 5’-TCGCGGTCATTCCCTCTAT-3’ | Forward |
| EGFRA-R | 5’-TCGTACCACTGAATCGTCTCC-3’ | Reverse |
| FSHRA-F | 5’-ATCTCGCTTCGGCATCTTTA-3’ | Forward |
| FSHRA-R | 5’-TTCCCATCGAAACTCAGTCC-3’ | Reverse |
| GJA3_F | 5’-ACGGTTATCGGCAAAGTCTG-3’ | Forward |
| GJA3_R | 5’-GTGAAGTCGGACTGCTCGTC-3’ | Reverse |
| GNAI2-F | 5’-CCATCTGCAACAACAAGTGG-3’ | Forward |
| GNAI2-R | 5’-GGTCCTCAAATTTGGTCTGG-3’ | Reverse |
| INSL3-F | 5’-AACCTGTGGGAACTCACGTC-3’ | Forward |
| INSL3-R | 5’-GGGTGTCTTTCTCCCTGTCA-3’ | Reverse |
| ITGB1_F | 5’-CAAAGCAAACACGCAGTCAT-3’ | Forward |
| ITGB1_R | 5’-TGGGTTCTCAATGTTTGCAC-3’ | Reverse |
| JUP_F1 | 5’-CTCACCAAACTGCTCAACGA-3’ | Forward |
| JUP_R | 5’-GTCACTGGTGTTCTGCATGG-3’ | Reverse |
| LHCGR-F | 5’-ACCTGTGCATGGGGCTCTAC-3’ | Forward |
| LHCGR-R | 5’-TTAGTGATGGTGTGCCAACG-3’ | Reverse |
| OXT-F | 5’-TCCCCAGAAACAGCTCACTG-3’ | Forward |
| OXT-R | 5’-TCCTTCCTCGTCACAGGACT-3’ | Reverse |
| OXTRA-F | 5’-TTGGCTTACATCGTCTGCTG-3’ | Forward |
| OXTRA-R | 5’-TTCTGCCTCAGATCCTGGAA-3’ | Reverse |
| OXTRB-F | 5’-TTTGTCCAGATGTGGTCTGC-3’ | Forward |
| OXTRB-R | 5’-CACTGCATCCAGTCATGGAA-3’ | Reverse |
| PARD3_F | 5’-CAACAGCAGGCTACACCAAA-3’ | Forward |
| PARD3_R | 5’-CTCCCCGAGGAAGAATGTT-3’ | Reverse |
| PLCB3-F | 5’-AAAAGAGAGCAGGTCCGACA-3’ | Forward |
| PLCB3-R | 5’-ACAGTGGCTGGTTCATGTCA-3’ | Reverse |
| PPP3CA-F | 5’-GGTGGCACTTCGAATCATAGA-3’ | Forward |
| PPP3CA-R | 5’-GAAGAGGTACCGTGTTGTCG-3’ | Reverse |
| PVR_F | 5’-GCTTCCGCTGCATATTTGAT-3’ | Forward |
| PVR_R | 5’-CCTCTCCGGGACACTGTACT-3’ | Reverse |
| RAB5C_F | 5’-GGCAAGTCCAGCCTGGTG-3’ | Forward |
| RAB5C_R | 5’-CTGTGGTATCGCTCCTGACC-3’ | Reverse |
| RLN1-F | 5’-ACTATGGGGTGAAGCTCTGC-3’ | Forward |
| RLN1-R | 5’-GTGTGCCGACTGTCCTCTG-3’ | Reverse |
| RLN3-F | 5’-CGTATGGGGTGAAGCTCTGT-3’ | Forward |
| RLN3-R | 5’-TGCGAATCTTAGGTCCCTGT-3’ | Reverse |
| RXFP1-F | 5’-ACATCGTGCTGAGGGTCTTC-3’ | Forward |
| RXFP1-R | 5’-GATGCCCATTAGTCCGTCTG-3’ | Reverse |
| RXFP2-F | 5’-GGCCAACAGGTTCTTCTTCA-3’ | Forward |
| RXFP2-R | 5’-TGTAAAGGATGGGGTTCAGG-3’ | Reverse |
| RXFP32-F | 5’-CAACATCCTTGGGAACTCCA-3’ | Forward |
| RXFP32-R | 5’-AGCCCGAGTGCACAGACTAT-3’ | Reverse |
| SCRIB-F | 5’-GAGCTTGCAGACGCTACTGA-3’ | Forward |
| SCRIB-R | 5’-ATCCTCCGTCTGGAACTTGA-3’ | Reverse |
| SNAI2-F | 5’-CACACCTTGCCTTGTGTTTG-3’ | Forward |
| SNAI2-R | 5’-TTGGAGCAGTTCTTGCATTG-3’ | Reverse |
| TJP2_F | 5’-GCGTGGGTAAAGAGAAGCAG-3’ | Forward |
| TJP2_R | 5’-CTCTTCAGGTCTTTCCTCTTCAG-3’ | Reverse |
| TMEM47_F | 5’-ATTTCTGGTCGCCCTGGT-3’ | Forward |
| TMEM47_R | 5’-ATTCGCAGGTTGATGCTCTC-3’ | Reverse |
| TUBA_F | 5’-GCATGCTGAGCAACACCA-3’ | Forward |
| TUBA_R | 5’-CACCCTCTTCCATACCCTCA-3’ | Reverse |
