# Supplementary material for: Gonadotropins differentially regulate testicular cell adhesion and junctional complexes during flatfish spermiogenesis through the oxytocin and relaxin signaling pathways
Source: Front Cell Dev Biol. 2025 Jun 2;13:1574690. doi: 10.3389/fcell.2025.1574690 (PMC12171224; doi:10.3389/fcell.2025.1574690)
Supplement: Supplementary file 6 [file Table2.docx]

**TABLE S2**

Oligonucleotide sequences used for the synthesis of ISH probes.

| **Name** | **DNA sequence** | **Direction** |
| --- | --- | --- |
| INSL3-ISH-F | 5’-AACCTGTGGGAACTCACGTC-3’ | Forward |
| INSL3-ISH-R | 5’-AATGCAACATGACTGGGACA-3’ | Reverse |
| OXTRA-ISH-F | 5’-TTCGCTGAGCACCACATAAG-3’ | Forward |
| OXTRA-ISH-R | 5’-GAAACATTCAGTTAAGTGCTCGAC-3’ | Reverse |
| OXTRB-ISH-F | 5’-CTGTGAAAAGCCAGCTGAAC-3’ | Forward |
| OXTRB-ISH-R | 5’-AAGCAGCCGCAATACTCTGT-3’ | Reverse |
| RLN1-ISH-F | 5’-CAGAGGACAGTCGGCACAC-3’ | Forward |
| RLN1-ISH-R | 5’-CTCAGCACAAGCGTCCAATA-3’ | Reverse |
| RLN3-ISH-F | 5’-ACAGGGACCTAAGATTCGCA-3’ | Forward |
| RLN3-ISH-R | 5’-TGGCAGCCCTTTCCATATAC-3’ | Reverse |
| RXFP1-ISH-F | 5’-GCGACCTTTCAAAGAGACCA-3’ | Forward |
| RXFP1-ISH-R | 5’-AATTGATTTGGCGGCAGAC-3’ | Reverse |
| RXFP2-ISH-F | 5’-AGGGAGCAGGTGGAACTCTT-3’ | Forward |
| RXFP2-ISH-R | 5’-TGTGTATAAATACATGAGAGGGAAG-3’ | Reverse |
| RXFP3-ISH-F | 5’-CTTCCCCCTGACTGTGTGTT-3’ | Forward |
| RXFP3-ISH-R | 5’-TGTTGTTCTCTTCAGTGCATTC-3’ | Reverse |
